# The *Eucalyptus grandis NBS-LRR* Gene Family: Physical Clustering and Expression Hotspots

**DOI:** 10.3389/fpls.2015.01238

**Published:** 2016-01-12

**Authors:** Nanette Christie, Peri A. Tobias, Sanushka Naidoo, Carsten Külheim

**Affiliations:** ^1^Department of Genetics, Forestry and Agricultural Biotechnology Institute, Genomics Research Institute, University of PretoriaPretoria, South Africa; ^2^Department of Plant and Food Sciences, Faculty of Agriculture and Environment, University of SydneyNSW, Australia; ^3^Research School of Biology, College of Medicine, Biology and Environment, Australian National UniversityCanberra, ACT, Australia

**Keywords:** *Eucalyptus grandis*, NB-ARC, *NBS-LRR*, resistance genes, gene clusters

## Abstract

*Eucalyptus grandis* is a commercially important hardwood species and is known to be susceptible to a number of pests and pathogens. Determining mechanisms of defense is therefore a research priority. The published genome for *E. grandis* has aided the identification of one important class of resistance (*R*) genes that incorporate nucleotide binding sites and leucine-rich repeat domains (NBS-LRR). Using an iterative search process we identified *NBS-LRR* gene models within the *E. grandis* genome. We characterized the gene models and identified their genomic arrangement. The gene expression patterns were examined in *E. grandis* clones, challenged with a fungal pathogen (*Chrysoporthe austroafricana*) and insect pest (*Leptocybe invasa*). One thousand two hundred and fifteen putative *NBS-LRR* coding sequences were located which aligned into two large classes, Toll or interleukin-1 receptor (*TIR*) and coiled-coil (*CC*) based on NB-ARC domains. *NBS-LRR* gene-rich regions were identified with 76% organized in clusters of three or more genes. A further 272 putative incomplete resistance genes were also identified. We determined that *E. grandis* has a higher ratio of *TIR* to *CC* classed genes compared to other woody plant species as well as a smaller percentage of single *NBS-LRR* genes. Transcriptome profiles indicated expression hotspots, within physical clusters, including expression of many incomplete genes. The clustering of putative *NBS-LRR* genes correlates with differential expression responses in resistant and susceptible plants indicating functional relevance for the physical arrangement of this gene family. This analysis of the repertoire and expression of *E. grandis* putative *NBS-LRR* genes provides an important resource for the identification of novel and functional *R*-genes; a key objective for strategies to enhance resilience.

## Introduction

*Eucalyptus grandis* W. Hill ex Maiden (Flooded Gum) is an Australian myrtaceous forest species that is grown for timber-related products in many parts of the world (Myburg et al., 2014). Eucalypts are susceptible to a range of co-evolved and exotic pests and pathogens (Whyte et al., 2011), which can inhibit growth, reduce production and have significant effects on local economies (Coutinho et al., 1998; FAO, 2010). Therefore, determining the genetic basis for resistance to biotic stress is an urgent research priority with the goal of developing resilient forestry. As an important commercial species, *E. grandis* was selected as one of the first woody plants for genome sequencing (Myburg et al., 2011). The annotated draft genome for *E. grandis*, from a 17 year old inbred clone, BRASUZ1 (genome size of 640 Mbp, 11 haploid chromosomes), was released in 2011 with more comprehensive annotation data published in 2014 (Myburg et al., 2014). The availability of genomic sequence data for *E. grandis* makes it a useful model plant for the study of defense responses to current and emerging pathogens across a range of species within the Myrtaceae family.

Current understanding of plant defense responses is largely derived from research on crop plants and model species. After preformed defenses, such as cutin, waxes (Kolattukudy, 1980), and cell walls, a first line of defense is recognition and response to common pathogen-associated molecular patterns (Monaghan and Zipfel, 2012). Plants also exhibit pest (Aggarwal et al., 2014) and pathogen-specific responses mediated by resistance (*R*) genes (Ellis et al., 2000). The recognition proteins, encoded by *R*-genes, are predominantly intracellular receptors with high target specificity. Recognition and subsequent nucleotide binding leads to a cascade of responses that disrupt pathogenicity (Bernoux et al., 2011). Host-specific pathogens are known to deliver effector molecules into plant cells to disrupt host defense and increase pathogen virulence (Voegele and Mendgen, 2003). Resistant host plants harbor specific recognition receptors to counter pathogen effectors and initiate downstream immune response in a gene-for-gene manner (Flor, 1971). Models for pathogen perception describe direct and indirect recognition, thereby directing contrasting *R*-gene evolution (Jones and Dangl, 2006). Direct recognition involves the binding of receptors to effectors with demonstrated evidence from *Linum usitatissimum* (flax) response to *Melampsora lini* (flax rust; Dodds et al., 2006). Indirect recognition, appears to be more common than direct effector recognition (Kaschani et al., 2010; Bhattacharjee et al., 2011), and involves recognition of effector modified host proteins in what is termed the “guard hypothesis” (Jones and Dangl, 2006). Recently the recognition of effector modified host decoy proteins was validated in *Arabidopsis thaliana* (Le Roux et al., 2015), confirming the extension of the guard hypothesis to include decoy strategies and suggesting important evolutionary responses in plants to evading pathogens (van der Hoorn and Kamoun, 2008). While direct recognition requires adaptive responses to fast evolving pathogens, thereby driving rapid *R*-gene evolution, indirect recognition involves the ongoing surveillance of host molecular integrity and has been shown to permit long-term maintenance of *R*-genes (Stahl et al., 1999). Several classes of *R*-genes have been identified, however the most abundant are characterized by a centrally located nucleotide-binding site (NBS) domain and a carboxy-terminus leucine rich repeat domain (LRR; DeYoung and Innes, 2006), the *NBS-LRR* gene family.

The NBS domain incorporates sub-domains that are highly conserved in NBS-LRR and are therefore likely to be good predictors of gene function (Tameling et al., 2006). Within this domain is the *NB*S P-loop structure, involved in adenosine triphosphate (ATP) hydrolysis (Walker et al., 1982), near the N-terminus, as well as two ARC domains [*A*PAF-1 (apoptotic protease activating factor-1), *R*-proteins and *C*ED-4 (*Caenorhabditis elegans* death-4 protein) van Ooijen et al., 2008], at the C-terminus (NB-ARC). The NB-ARC domain is known to be involved in activating the hypersensitive response (HR), an important plant defense strategy characterized by rapid programmed cell death of affected and surrounding cells, thereby blocking disease progression of biotrophic and hemi-biotrophic pathogens (Mur et al., 2008). The LRR protein motif occurs across organisms and the alpha/beta horseshoe fold is believed to be involved in protein-protein interactions (Matsushima and Miyashita, 2012). This predicted feature, as well as the observed diversifying selection (Michelmore and Meyers, 1998) in this region of plant NBS-LRRs, suggested a role in pathogen recognition. Evidence supporting this is scant however and therefore their function in many plants is still unclear (DeYoung and Innes, 2006). Other structures often present are the coiled-coil (CC) motif and the Toll or interleukin-1 receptor homology domain (TIR) at the amino-terminus (McHale et al., 2006). Recent evidence points to these regions having strategic roles in pathogen recognition and signaling (Chang et al., 2013; Williams et al., 2014). These fused domains, and chimeric variants of them, commonly occur across plant genomes, although the TIR domain is notably absent from most monocotyledons (Tarr and Alexander, 2009).

The published genome of *E. grandis* has permitted investigations into important gene families such as terpene synthases (Külheim et al., 2015), lignin biosynthesis genes (Carocha et al., 2015), and pathogenesis-related genes (Naidoo et al., 2014). These analyses indicate that *E. grandis* has expanded gene families in relation to other plants. Further insights have been the high representation of defense-related genes amongst recent domain expansions and tandem duplications (Kersting et al., 2015). Themes that emerge for *NBS-LRR* genes across other woody plant genomes include the clear evolutionary divergence between two the major classes (TIR and CC), high proportions of genomic clustering and the large numbers within this gene family, >1% of protein coding genes (Tobias and Guest, 2014).

Here we determine the potential gene complement, physical location and genomic arrangement of *NBS-LRR* genes within the *E. grandis* genome. We classify these genes based on amino acid sequences of conserved domains. Further to this, we take two serious biotic stressors of *E. grandis*, stem canker (*C. austroafricana*), and the *Eucalyptus* gall wasp (*Leptocybe invasa*) and review *NBS-LRR* gene expression from resistant and susceptible clones. Apart from the characterization of all potential complete and partial *NBS-LRR* genes, key questions addressed in this research are; (i) what does the physical arrangement for this gene family indicate, (ii) can the putative genes be validated by expression data, and (iii) are there patterns of *NBS-LRR* gene expression under biotic stress?

## Materials and methods

### Identification of putative *NBS-LRR* genes

**Figure 1:**
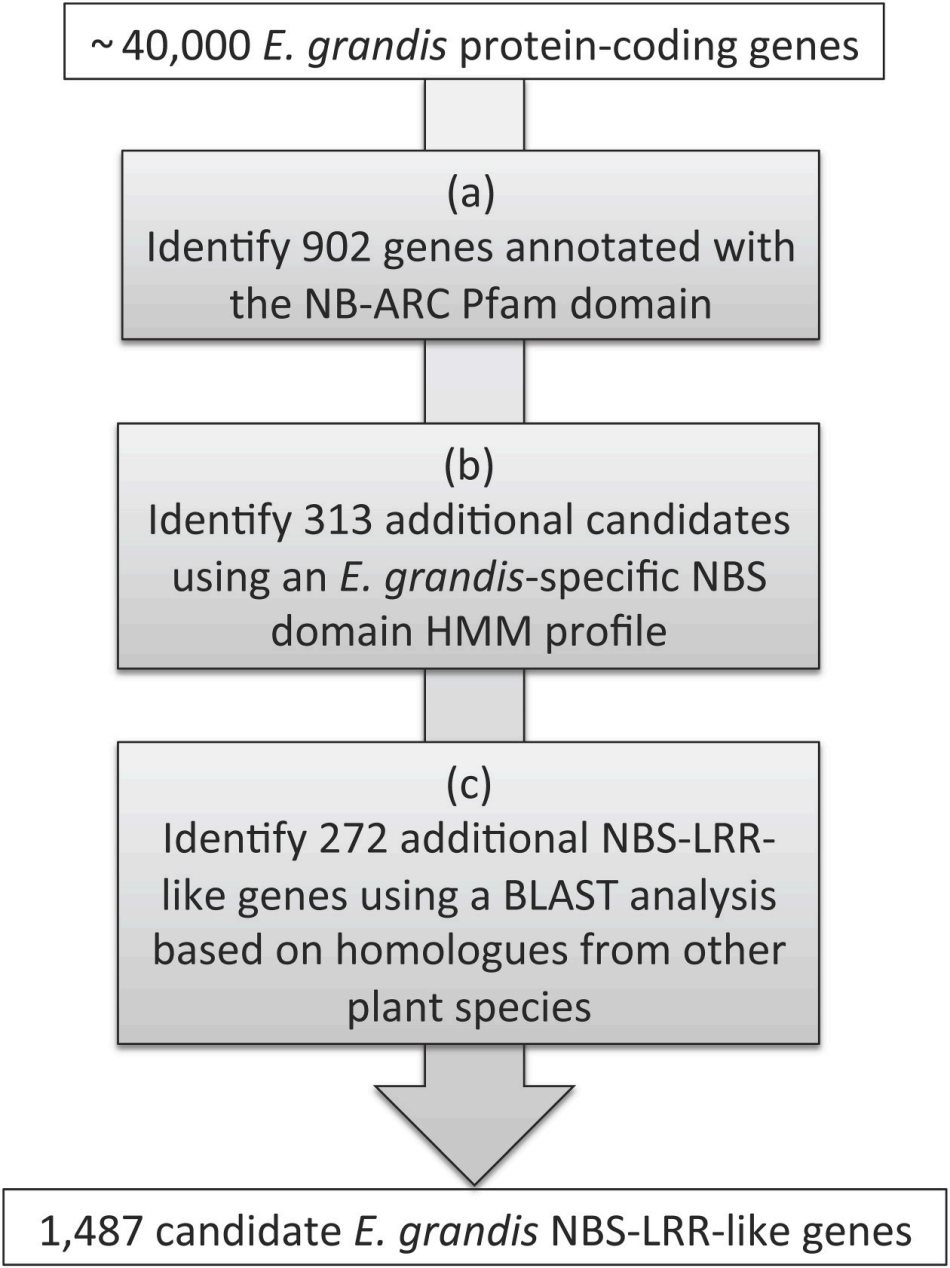
**Flow diagram of the strategy (a–c) that was followed to identify putative ***NBS-LRR*** genes within the ***Eucalyptus grandis*** genome**.

The *E. grandis* annotation information file that was released as part of Phytozome v7.0 (Egrandis_162_annotation_info.txt, http://phytozome.jgi.doe.gov) was used to identify an initial list of 902 NB-ARC Pfam domain (PF00931) containing proteins (Goodstein et al., 2012). The presence of the NBS domain in each of the identified protein sequences was confirmed with PfamScan (www.ebi.ac.uk/Tools/pfa/pfamscan/; *e* < 1e-04; Figure 1a). An *E. grandis* specific Hidden Markov Model (HMM) for the conserved NB-ARC protein domain was constructed with 150 sequences (PfamScan *e* < 1e-60) using HMMER (Eddy, 2010). The *E. grandis* specific HMM was used to search for additional NB-ARC domains within the *E. grandis* protein sequence data using hmmsearch (*e* < 1e-04; Figure 1b). As a complement to this list, a nucleotide search was conducted using Basic Local Alignment Search Tool (BLAST) in Phytozome with three *NBS-LRR* genes from distantly related species (*A. thaliana* gi|3309618|, *Medicago truncatula* gi|357495078|and *Solanum tuberosum* gi|323370546|). The BLAST search identified genes with expected match results (*e*-value) of < 1e-10 and alignments >500 bp. *E. grandis* peptide homologs, from the results of this search, were then extracted and included in the potential *NBS-LRR* gene list (Figure 1c). The final list contained gene models that incorporated both complete and partial *NBS-LRR* genes as well as genes that shared homology with disease resistance genes from other plant species but without a full NB-ARC domain. The total list (1487 sequences) of candidate *NBS-LRR* genes was used in physical cluster and expression analysis while a reduced list (1215 sequences), including only genes with full NB-ARC domains, was used in all other downstream analysis.

### Identification of conserved motifs

PfamScan was used to determine, from the library of Pfam HMMs, whether the identified candidate sequences encoded NB-ARC, TIR and LRR domains. Additionally, sequences were identified as having CC motifs according to COILS software (Lupas et al., 1991; http://www.ch.embnet.org/software/COILS_form.html), with a threshold of 0.9. Further validation was conducted with Paircoil2 (McDonnell et al., 2006; http://paircoil2.csail.mit.edu/; *p* < 0.025). MEME version 4.10.1 (Multiple Expectation Maximization for Motif Elicitation; http://meme-suite.org/tools/meme; Bailey and Elkan, 1994) analysis was used to identify conserved motifs within CC-NBS-LRR (CNL) and TIR-NBS-LRR (TNL) classes. The NB-ARC and TIR domain amino acid sequences, extracted from full protein sequences of 132 CNL and 174 TNL, were independently examined. A maximum of 15 motifs was allowed with zero or one motif per sequence.

### Alignment and phylogenetic analysis

Alignment and phylogenetic relationships for NB-ARC domains (extracted from full protein sequences) were conducted using Mega6 (Tamura et al., 2013). The analysis was based on 480 *E. grandis* NBS-LRRs [154 TNLs, 123 CNLs and 203 NBS-LRRs (NLs)] and 15 NB-ARC sequences from outlier species including *A. thaliana* (At5g66900, At4g26090, At3g46530, At5g43470, At5g45260, At5g45250, At1g27170, At1g72840, At4g16950, At5g46520, At3g44630, At1g56510, At5G40060; Meyers et al., 2003), *Malus domestica* (AFP82245.1) and *Ktedonobacter racemifer* (WP_007911379.1; **Figure 3A**; Figure S1). Phylogenies for all NB-ARC domains from TIR [240 TIR-NBS (TN), 2 TIR-CC-NBS (TCN), 154 TNL] and non-TIR [86 CC-NBS (CN), 123 CNL, 202 NBS (N), 204 NL, 1 BED *zinc* finger-CNL (BCNL)] derived gene models were also constructed (Figures S2, S3). Due to pairwise distance calculation problems, 55 TIR and 153 non-TIR sequences were removed from the analysis. The sequences were aligned using ClustalW (Thompson et al., 1994) with default parameters.

### Physical cluster analysis

On reviewing the literature we determined an appropriate definition for gene clusters and superclusters that permitted the mapping and analysis of *E. grandis* putative *NBS-LRR* genes (Meyers et al., 2003; Kohler et al., 2008; Jupe et al., 2012). We defined a gene cluster as a genomic region containing at least three *NBS-LRR* genes, (i) with < 9 other genes between neighboring *NBS-LRR* genes and (ii) in which two neighboring *NBS-LRR* genes are < 250 kb apart (example Figure S4A). We defined a gene supercluster as a genomic region containing at least one *NBS-LRR* gene cluster and at least two additional *NBS-LRR* genes, (i) with < 99 other genes between neighboring *NBS-LRR* genes and (ii) in which two neighboring *NBS-LRR* genes are < 2500 kb (2.5 Mb) apart (example Figure S4B).

To test the validity of the supercluster definition, we determined the probability of randomly observing at least one cluster and two additional genes (three NBS-LRR clusters/genes) within 5 Mb of the *E. grandis* genome (640 Mb). However, the calculation, using a permutation approach (R script) as described below, assumed the clusters and genes (contributing to supercluster) were randomly distributed across the *E. grandis* genome (640 Mb), whereas this gene family are found in clusters. The approach did not take into account the span of clusters, on average 400 kb. The permutation test: (1) Distribute 535 clusters/genes (136 clusters + 399 singletons) randomly across 640 Mb (640,000 kb), (2) Calculate the number of times that a group of three clusters/genes span < 5000 kb and divide the result by the number of groups (535), (3) Repeat the previous two steps 1000 times and save the resulting probability every time, (4) Calculate the 95th percentile of the resulting distribution of probabilities. The probability of observing three NBS-LRR clusters/genes within 5 Mb for a random distribution of the 535 clusters/genes over the 640Mb is 41%.

A cluster was defined as CNL-type if no genes with TIR domains were present, but at least one gene had a CC motif. Clusters were defined as TNL-type if no genes with CC motifs, but at least one gene had a TIR domain. Mixed-type clusters have at least one gene with a CC motif and one gene with a TIR domain present, while other-type clusters have no genes with CC motifs or TIR domains [therefore only genes in classes leucine rich repeat (L), N and NL].

### Visualization of *NBS-LRR* genes on chromosomes

The positions of the TN(L), CN(L), and N(L) classes of the NBS-LRR genes were visualized using Circos (Krzywinski et al., 2009). Gene models, physical clusters and superclusters were mapped to the 11 *E. grandis* chromosomes using base pair start positions in Mapchart 2.2 (Voorrips, 1994).

### NBS-LRR predicted protein structure

Peptide sequences of a representative predicted gene from TNL (Eucgr.C00020) and non-TNL (Eucgr.L01363) were submitted to the I-Tasser server (http://zhanglab.ccmb.med.umich.edu/I-TASSER/) to determine predicted protein structures.

### *NBS-LRR* gene numbers in other plants

A comparative review of *NBS-LRR* gene predictions from sequenced plants was undertaken using Phytozome genome data and published *NBS-LRR* papers for; *A. thaliana* (Meyers et al., 2003), *Oryza sativa* (Zhou et al., 2004), *Populus trichocarpa* (Kohler et al., 2008), *Hevea brasiliensis* (Rahman et al., 2013), *Malus* × *domestica* (Arya et al., 2014), *Solanum lycopersicum* (Sato et al., 2012), *Threobroma cacao* (Argout et al., 2011), *Manihot esculenta* (Lozano et al., 2015), *Vitis vinifera* (Argout et al., 2011 suppl.) and *S. tuberosum* (Jupe et al., 2012). Numbers of complete (CNL, TNL, and NL classes) and incomplete *NBS-LRR* gene models (CN, TN, N classes) were graphed (**Figure 5**).

### Biotic stress trials

Clones of *E. grandis* were challenged with the fungal stem canker pathogen (*C. austroafricana*) and leaf gall wasp (*L. invasa*) as described by Mangwanda et al. (2015) and Oates et al. (2015), respectively. Infected *C*. *austroafricana* stem samples were collected 3 days post inoculation and infested *L. invasa* leaf samples were collected 7 days post infestation. Total RNA was extracted and sent to the Beijing Genome Institute (BGI) for RNA-Sequencing using the Illumina Genome Analyzer with a 50 bp paired end module (Illumina, San Diego, CA).

### RNA-Seq data analysis

RNA-Seq data was analyzed using the Galaxy workspace (Giardine et al., 2005; Blankenberg et al., 2010; Goecks et al., 2010). FASTQC v0.52 was used to verify RNA-Seq data quality. Reads were mapped to the *E. grandis* v1.1 genome assembly using Bowtie (Langmead et al., 2009) and Tophat2 v2.0.9 (Trapnell et al., 2009). Mapping was allowed over multiple domains, however the default Tophat setting discards reads mapped to more than 20 locations. Therefore, if more than 20 family members existed with identical domains, mapping to those domains would have been unlikely. We used a mix of reads (unique and multiple with < 20 family members) but applied fragment bias correction and multi read mapping correction in Cuffdiff (Trapnell et al., 2010; Roberts et al., 2011). The expression quantification step would have corrected for this multiple mapping to highly conserved domains based on unique reads mapped to other regions of the expressed transcripts. It is therefore expected that highly expressed genes would be accurately mapped. Although genes with low numbers of mapped reads may represent low expression of those genes or may indicate false mapping, the depth of sequencing in our experiments (36–40 million reads per sample) would have improved detection of lowly expressed genes.

Mapped reads were assembled into transcripts and fragments per kilobase of exon per million fragments mapped (FPKM) values were calculated with Cufflinks v2.1.1 (Trapnell et al., 2010). Quartile normalization was conducted in Cufflinks. Detailed analysis was as described by Mangwanda et al. (2015) and Oates et al. (2015). The Eucalyptus data sets supporting these results are available in the NCBI Gene Expression Omnibus repository for *C. austroafricana* challenge (GSE67554) and NCBI BioProject ID PRJNA305347 for *L. invasa* challenge.

### *NBS-LRR* transcript expression analysis

Expression profiles for the putative *NBS-LRR* genes were extracted from a transcriptome-wide expression matrix using a custom Python script. Expression data were only available for genes in *E. grandis* v1.1. Analysis of variance (ANOVA) for putative *NBS-LRR* genes from treatment, control, resistant and susceptible groups was performed in GenStat (v. 16.2.0.11713, VSN International, Hemel Hempstead, UK). The *E. grandis* datasets from both *C. austroafricana* and *L. invasa* were analyzed separately. Expression analysis was based on the log_2_ fold change of inoculated vs. control samples. Genes in resistant and susceptible plants were considered up or down-regulated if their log_2_ gene expression ratios were >1 or smaller than −1. Differential expression was determined by taking significant *p*-values (< 0.01) from the ANOVA analysis by treatment and comparing this data with fold change values. Heatmaps that depict gene expression [as log_2_ of the normalized read count (FPKM)] and accompanying hierarchical clustering of both gene expression and treatments, were drawn in R studio (v. 0.98.981, RStudio Team, 2015) using the gplots and RColorBrewer packages. Color breaks, using red, yellow and green, were non-linear and adjusted individually to each heatmap (e.g., a subsample of *NBS-LRR* genes that had a maximum log_2_ FPKM value of 2 had color breaks at 0.1, 0.5, and 2).

A locus for resistance to *Puccinia psidii* (myrtle rust) has been mapped on the Eucalyptus reference map (Mamani et al., 2010). EMBRA125, one of the flanking markers of the *P. psidii* rust resistance (Ppr1) locus is closely linked to marker ePT_640786 (Kullan et al., 2012). This marker sequence has an estimated position of 52,900,000 bp on chromosome 3 of the *E. grandis* reference genome sequence and overlaps with the position of supercluster C-3. We determine the expression within this cluster in response to *C. austroafricana* (see heatmap **Figure 6D**).

## Results

### Identification of putative *NBS-LRR* genes

**Figure 2:**
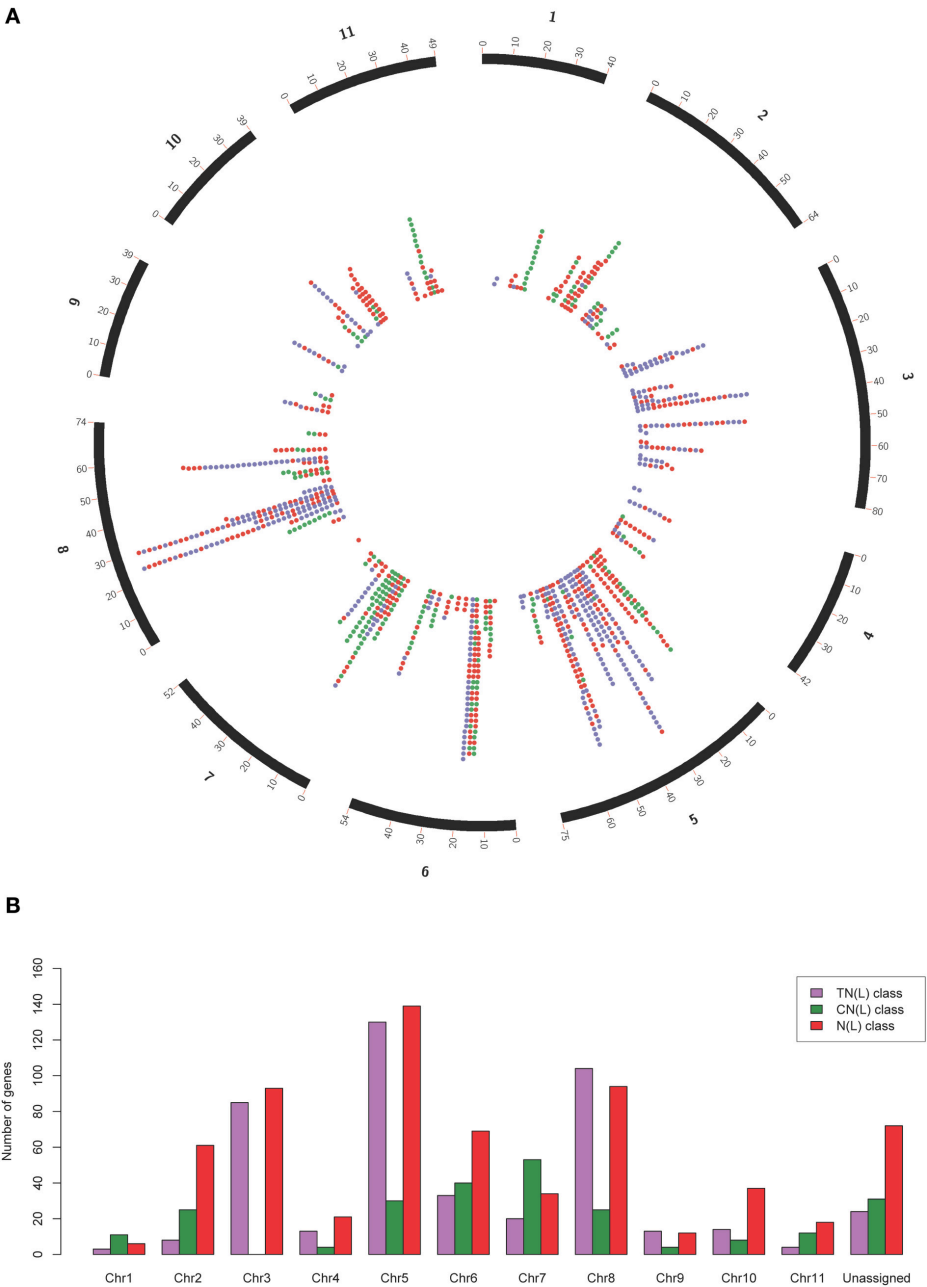
**Circos plot (A) and class distribution (B) of the positions and numbers of complete and partial ***NBS-LRR*** genes (total = 1215) per chromosome identified within the ***Eucalyptus grandis*** genome**. In the circos plot, purple dots represent genes in the TN(L) class, green dots represent genes in the CN(L) class and red dots represent genes in the N(L) class.

Our search strategy identified 1487 putative *NBS-LRR* genes within the *E. grandis* genome (Table 1). Of these, 557 gene models appeared to be complete, incorporating both the NB-ARC and leucine-rich repeat (LRR) domains, and 660 additional gene models incorporated the NB-ARC domain but lacked LRRs (partial *NBS-LRR*). Figure 2 gives the positional and class distribution per chromosome of all the identified *NBS-LRR* genes with a full NB-ARC domain. Gene models with domain chimeras were identified (Table S1) including one BED *zinc* finger-CNL, one CTN and two TCN as well as many with duplicate or variant fused domains (112 sequences) such as CNNL, CNN, NNL, NLNL, NNLN, NN, TNNL, TNN, TNTN, TTN. Incomplete gene models (270) were also identified that were homologs of disease resistance genes from other plant species (Table 1).

**Table 1:** **The full set of nucleotide-binding site leucine-rich repeat (NBS-LRR) gene models, including complete, partial and incomplete genes, identified in the ***Eucalyptus grandis*** genome**.

**Set**	**Number**	**%**	**Set**	**Number**	**%**	**Set**	**Number**	**%**	**Class**	**Number**	**%**	**Identification**	**Number**	**%**
NBS LRR-like genes	1487	100	With N	1217	81.8	With Nand L	557	37.5	TNL	174	11.7	Pfam NB-ARC	162	10.9
												Egr-specific HMM	12	0.8
									CNL	133	8.9	Pfam NB-ARC	128	8.6
												Egr-specific HMM	5	0.3
									NL	250	16.8	Pfam NB-ARC	187	12.6
												Egr-specific HMM	63	4.2
						With N, without L	660	44.4	TN	276	18.6	Pfam NB-ARC	214	14.4
												Egr-specific HMM	61	4.1
												BLAST homolog	1	0.1
									CN	107	7.2	Pfam NB-ARC	76	5.1
												Egr-specific HMM	31	2.1
									N	277	18.6	Pfam NB-ARC	135	9.1
												Egr-specific HMM	141	9.5
												BLAST homolog	1	0.1
			Without N	270	18.2	With L, without N	133	8.9	TL	1	0.1	BLAST homolog	1	0.1
									CL	3	0.2	BLAST homolog	3	0.2
									L	129	8.7	BLAST homolog	129	8.7
						Without N or L	137	9.2	T	79	5.3	BLAST homolog	79	5.3
									C	4	0.3	BLAST homolog	4	0.3
									None	54	3.6	BLAST homolog	54	3.6

*N, NB-ARC domain; L, Leucine-rich repeat domain; T, Toll or interleukin-1 receptor domain; C, Coiled-coil domain; Egr, Eucalyptus grandis; HMM, Hidden Markov model; BLAST, Basic local alignment search tool*.

### Domain validation

The two major categories of putative *NBS-LRR* genes include sequences from the CNL class (which incorporate the amino acid tryptophan within the kinase 2 sub-domain) and sequences from the TNL class. The CNL class, identified as sequences containing the CC motif according to COILS, consisted of 132 complete *NBS-LRR* gene models. However, only 81 of these could be confirmed with Paircoil2 (*p* < 0.025). The TNL class consisted of 174 complete *NBS-LRR* gene models containing an N-terminal TIR domain. Out of the sequences in the NL class, 88 were physically located within CNL-type clusters and 107 within TNL-type clusters (Table S1). MEME analysis of NB-ARC domains from the mentioned classes [CNL, TNL, NL (within CNL-type clusters) and NL (within TNL-type clusters)] identified conserved motifs with homology to *A. thaliana* motifs (as identified in Meyers et al., 2003; Table S2). MEME analysis also identified motifs for TIR domains within all TN(L) containing sequences (Table S2).

### Phylogenetic analysis

**Figure 3:**
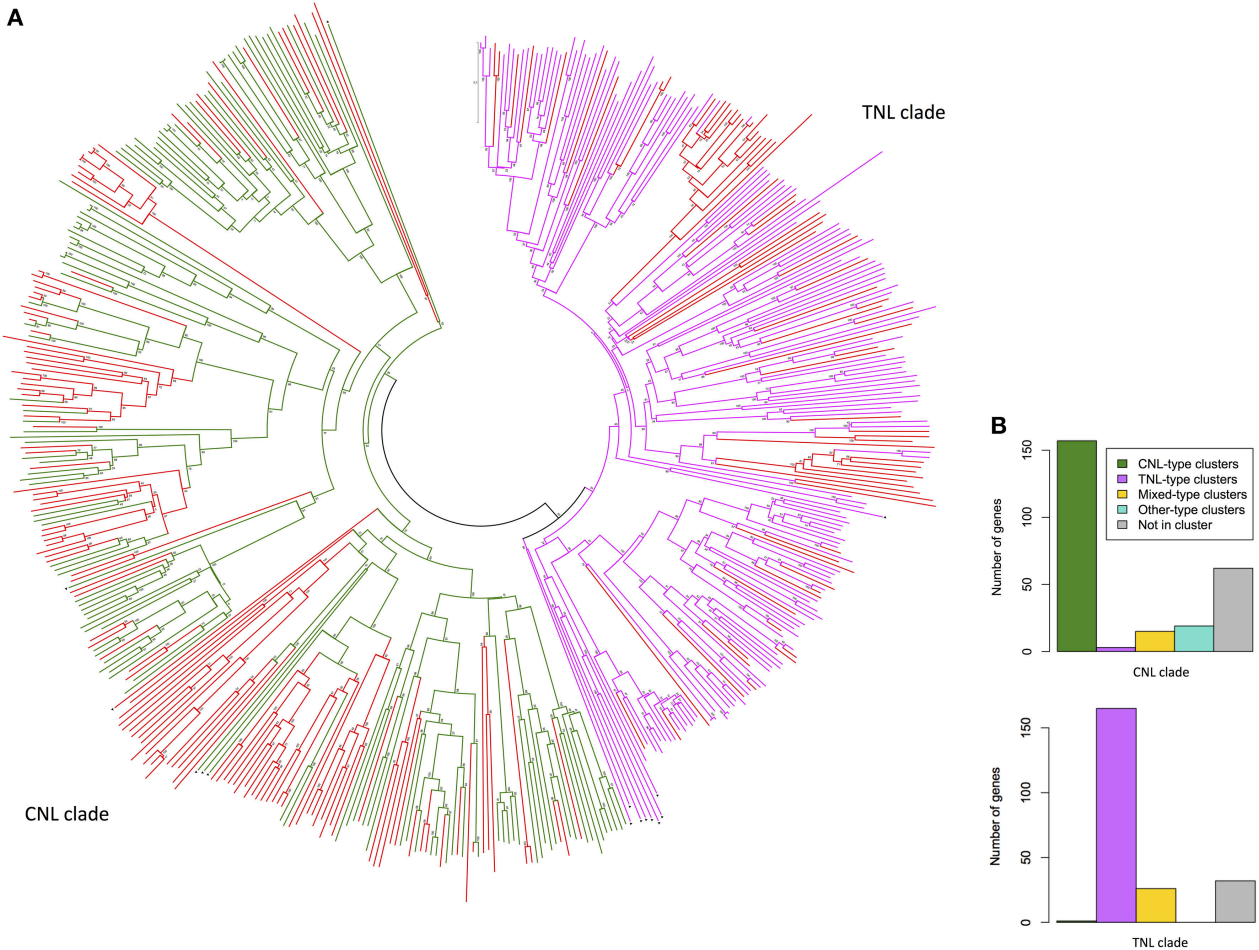
**(A)** Evolutionary relationships of *Eucalyptus grandis* NB-ARC domains from putative NBS-LRR genes. The evolutionary history was inferred using the Neighbor-Joining method. The optimal tree with the sum of branch length = 69.73913604 is shown. The tree is drawn to scale, with branch lengths in the same units as those of the evolutionary distances used to infer the phylogenetic tree. The evolutionary distances were computed using the p-distance method and are in the units of the number of amino acid differences per site. The analysis involved 495 amino acid sequences (480 *E. grandis*). All ambiguous positions were removed for each sequence pair. There were a total of 1089 positions in the final dataset. Scale: 0.1 substitutions per site. Evolutionary analyses were conducted in MEGA6 (Tamura et al., 2013). (NL = red, CNL = green, TNL = pink, black triangle denotes outlier NB-ARC domains from other species). **(B)** Cluster type distribution of *E. grandis NBS-LRR* genes for the two major phylogenetic clades.

The dataset of 557 complete *NBS-LRR* gene models was reduced to include all sequences for which pairwise distances between NB-ARC domain sequences could be calculated and for which only single NB-ARC domains were present. The evolutionary relatedness of 480 NB-ARC domains (123 CNL, 154 TNL, 203 NL) from complete *NBS-LRR* gene models separated into the two major clades: *CNL* and *TNL* (Figure 3A). Of the 203 NL sequences, 70 aligned with TNL and 133 with CNL NB-ARCs. The comparative numbers of gene models that aligned with TNL and CNL structures were 47% (224) and 53% (256), respectively. As expected, most of the genes within the CNL phylogenetic clade (61%) were physically located in CNL-type clusters, whereas only three genes (1%) were in TNL-type clusters (Figure 3B). Similarly, most genes within the TNL clade (74%) were physically located in TNL-type clusters, whereas one gene was in a CNL-type cluster (0.045%; Figure 3B).

While generally the phylogenetic clustering of putative *NBS-LRR* genes supported recently evolved and syntenic duplications, on chromosomes, we also found occurrences of highly similar sequences from different chromosomes. Examples we identified included five similar CNL-like genes (Eucgr.F03324/Eucgr.E02964, Eucgr.J02649/ Eucgr.G00249, Eucgr.H05030/Eucgr.G00087, Eucgr.G00250/Eucgr.J02651, Eucgr. J02654/Eucgr.G00259) and four similar TNL-like genes (Eucgr.J02654/Eucgr. G00259, Eucgr.F04314/Eucgr.C02650, Eucgr.E01971/Eucgr.D01584, Eucgr.E01990/ Eucgr.D01592; Figure S1). Recent and close phylogenetic clustering from different chromosomes was also evident for partial (TN and CN) sequences (Figures S2, 3).

### Cluster and supercluster analysis

**Figure 4:**
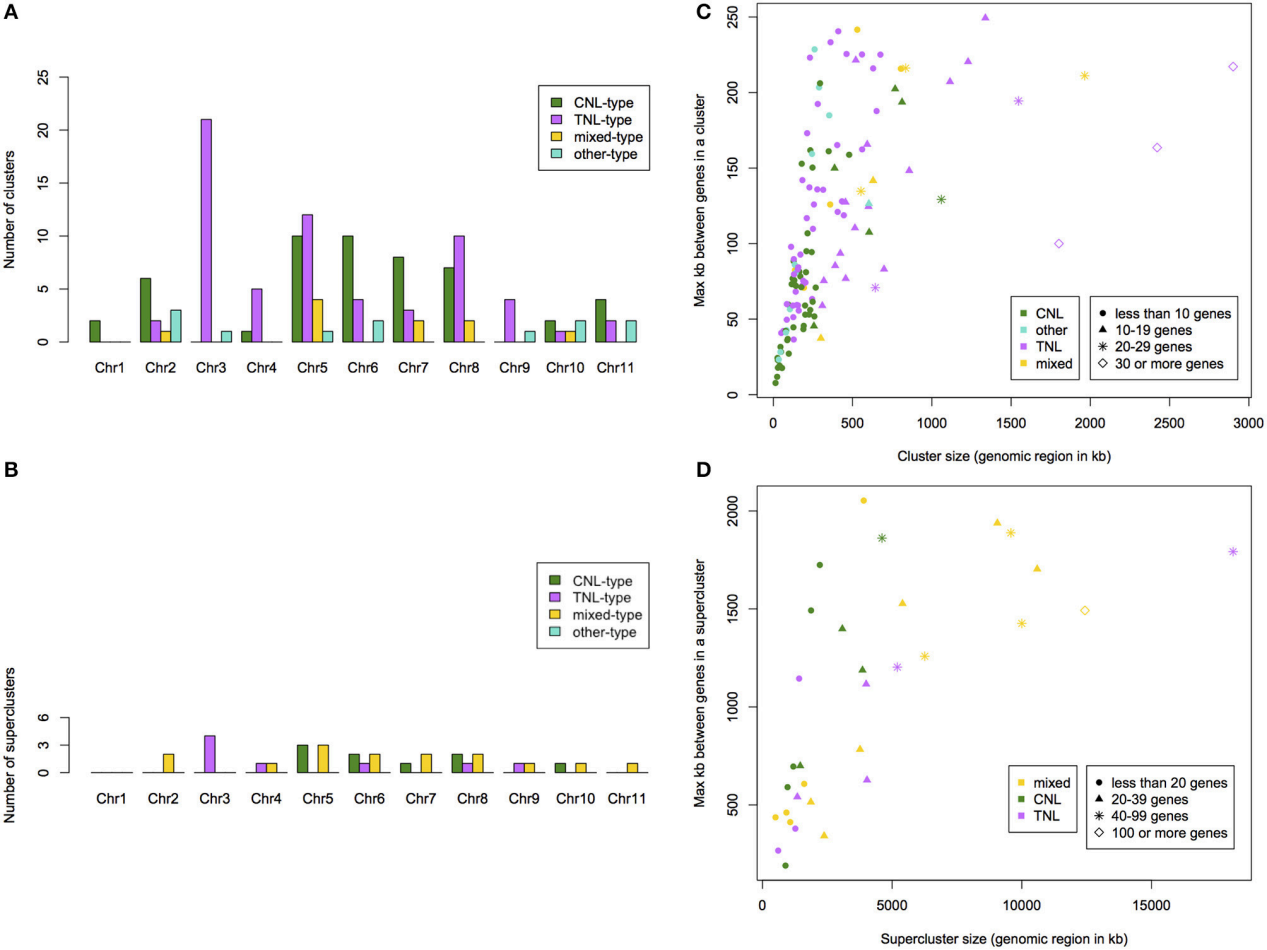
**Summary per chromosome of the number of identified ***NBS-LRR*** gene clusters (A) and superclusters (B) within ***Eucalyptus grandis*****. Cluster **(C)** and supercluster **(D)** size (physical size and number of genes) and type of cluster relative to the maximum distance between genes in a cluster within the *E. grandis* genome.

We determined that 76% of putative *NBS-LRR* genes occurred within clusters and 71% within superclusters (Table S1). We identified 136 *NBS-LRR* gene clusters (on average 12 per chromosome) with an average of 8 genes per cluster. Clusters span regions from 13 to 2900 kb. We identified 32 *NBS-LRR* gene superclusters with an average of 33 genes. Superclusters span regions from 0.5 up to 18 Mb. TNL-type clusters occurred predominantly on chromosomes 3, 5, and 8 (21, 12, and 10 clusters, respectively; Figure 4A). CNL-type clusters were more evenly distributed with the largest superclusters on chromosomes 5, 6, 7, and 8 (10, 10, 8, and 7 clusters, respectively; Figure 4B). Out of the 20% of clusters that span the smallest genomic region, 63% were CNL-type while the 20% of clusters spanning the largest genomic region, 63% were TNL-type (Figure 4C). The largest TNL-type supercluster (SC-C-2), also the cluster spanning the largest genomic region (Figure 4D), is on chromosome 3 and consists of 93 genes.

### Physical map of the 11 *E. grandis* chromosomes

Mapped chromosomal locations for all putative *NBS-LRR* genes, clusters and superclusters support the dense clustering for this gene family (Figure S5). Interestingly, though more TNL-like genes are present on chromosome 5 (Figure 2), a larger number of TNL-type clusters and superclusters are present on chromosome 3 (Figures 4A,B).

### NBS-LRR predicted protein structure

TNL (Eucgr.C00020) and CNL (Eucgr.L01363) protein structures were predicted using I-Tasser three dimensional modeling (Figure S6). The horseshoe fold of the LRR domain is indicated in the models. The GKT (Kinase 1) conserved subdomain is important in ATP hydrolysis while the hDD subdomain (Kinase 2) has a role in coordinating Mg^2+^ as a co-factor (Tameling et al., 2006). These two important sub-domains of NB-ARC, sometimes termed the Walker A and Walker B motifs (Walker et al., 1982), are identified within the models and designated A and B, respectively (Figure S6).

### Comparison of *NBS-LRR* gene copy numbers across other plants

**Figure 5:**
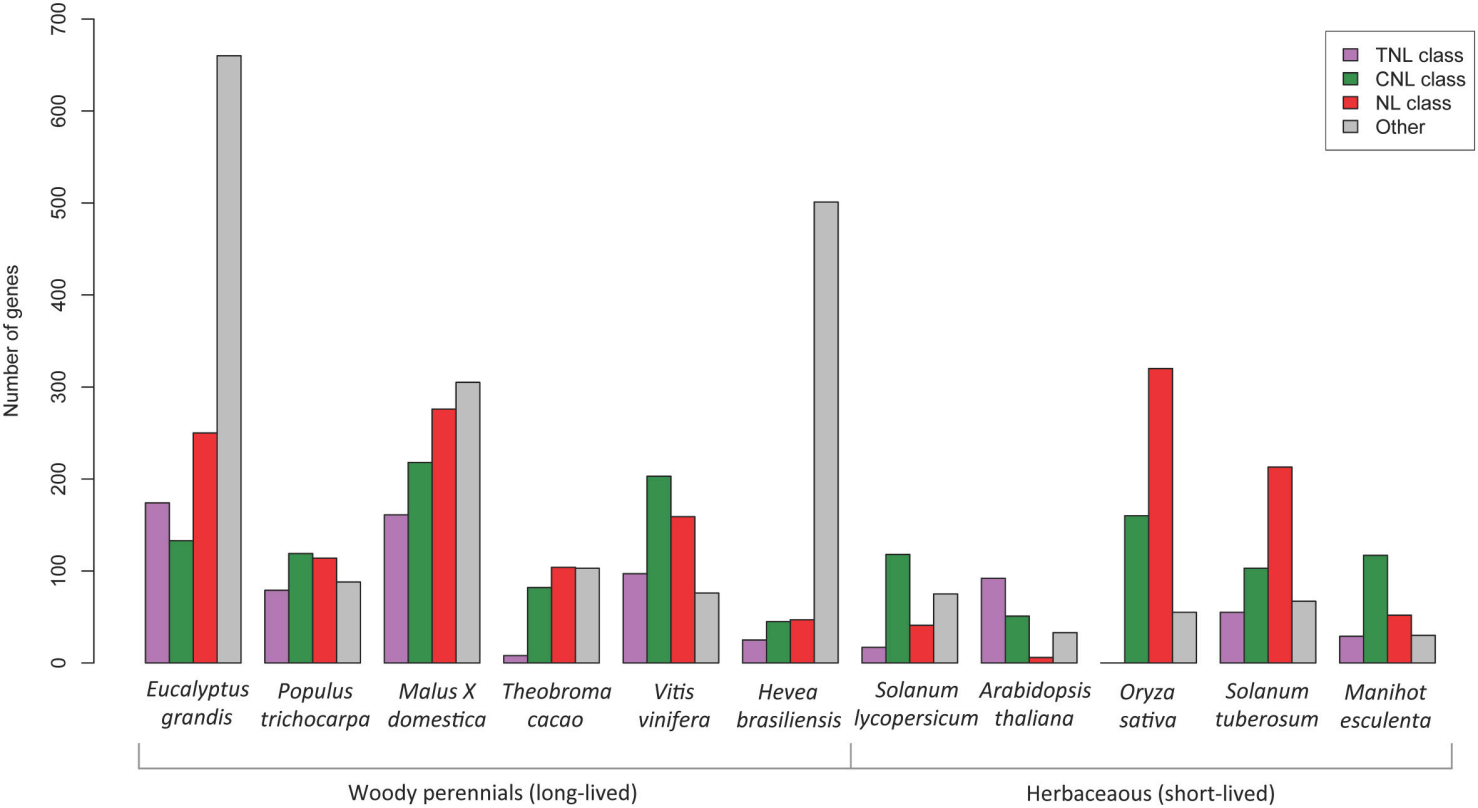
**Comparison of putative ***NBS-LRR*** gene numbers determined in the genomes of different plant species**. The “other” category (gray bars) include gene models in the TN, CN, and N classes.

When comparing the putative *NBS-LRR* gene results across other plant species (Figure 5) we found that the *E. grandis* genome harbored proportionately more putative TNL genes than all other species excepting *A. thaliana*. Interestingly, gene models that did not conform to the recognized structure of fused NB-ARC and LRR domains made up a large proportion of the total numbers of putative *NBS-LRR* genes within *E. grandis* and *H. brasiliensis*. Also, within the complete gene models, all plants excepting *A. thaliana*, appear to have large numbers of putative NL genes (lacking the amino terminal motif). *E. grandis, M. domestica, Theobroma cacao, S. tuberosum*, and *O. sativ*a all had greater proportions of NL compared to putative CNL or TNL genes. The comparisons of gene numbers should, however, be treated with caution as they relate specifically to the version of genome available during the time of study and the search strategy employed.

### Gene expression

Gene expression was detected for 1037 and 1047 complete, partial and incomplete putative NBS-LRR genes in the C. austroafricana and L. invasa studies, respectively (Table S1). Of these, 1027 genes were shared between the two transcriptomes while 10 and 20 were unique to C. austroafricana and L. invasa studies, respectively. Of the predicted 557 complete NBS-LRR genes, expression was measured for 423 within both transcript sets. Predicted full sequences that were not expressed included 21 CNL, 59 NL, and 54 TNL genes (Table S1).

Differential expression (DE) of putative *NBS-LRR* genes was evident under the two treatments (Table 2). Of the differentially expressed transcripts, 6 and 1 were significantly up-regulated only in resistant plants in the *C. austroafricana* and *L. invasa* studies, respectively (ANOVA treatment; *p* < 0.01; Table S1). The single significantly up-regulated gene after *L. invasa* challenge was an incomplete gene with only a putative TIR domain (Eucgr.H02120). Up-regulated genes from *C. austroafricana* challenge included two incomplete genes with only a putative TIR domain (Eucgr.L01982, Eucgr.F03897), an incomplete gene model homologous to TIR-NBS-LRR (Eucgr.H03831), an incomplete gene with only a putative NB-ARC domain (Eucgr.H03112; notably both Eucgr.H03831 and Eucgr.H03112 were significantly down-regulated in susceptible plants), an incomplete gene with only a putative TIR and NB-ARC domain (Eucgr.E02315) and a putative complete NBS-LRR that aligned within the CNL clade (Eucgr.B00924; Table S1). Both biotic challenges involved significant down-regulation of genes with 53 and 15 gene models significantly DE only in resistant and 39 and 13 DE only in susceptible *C. austroafricana* and *L. invasa* studies, respectively.

**Table 2:** **Summary of significantly differentially expressed (DE) nucleotide-binding site leucine-rich repeat (***NBS-LRR***) genes under biotic stress challenge in resistant (R) and susceptible (S) ***Eucalyptus grandis*** genotypes**.

**Category**	**Number of DE genes under *Leptocybe invasa* challenge**	**Number of DE genes under *Chrysoporthe austroafricana* challenge**
Up-regulated in R, up-regulated in S	0	11
Up-regulated in R, no change in S	23	68
Up-regulated in R, down-regulated in S	1	7
No change in R, up-regulated in S	12	18
No change in R, down-regulated in S	74	89
Down-regulated in R, up-regulated in S	2	4
Down-regulated in R, no change in S	77	105
Down-regulated in R, down-regulated in S	29	41

We also identified the differential lack of transcripts for putative genes from resistant and susceptible plants. For the *C. austroafricana* challenge we found 26 gene transcripts lacking in susceptible plants and 11 in resistant plants. In the *L. invasa* challenge we found 23 gene transcripts lacking in susceptible plants and 49 in resistant plants (Table S1).

**Figure 6:**
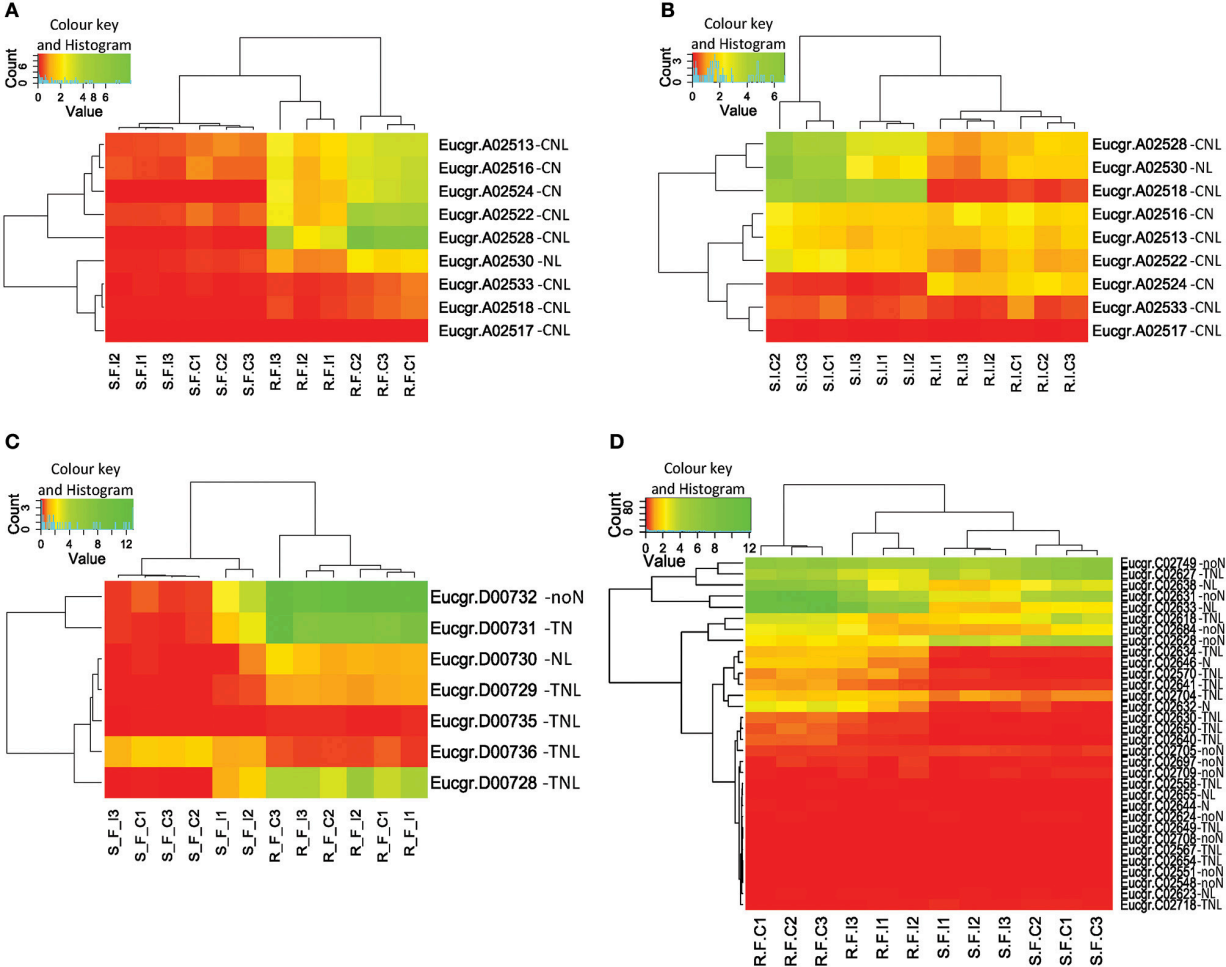
**Heatmaps and dendrograms of putative ***NBS-LRR*** gene expression for ***Eucalyptus grandis*** clones**. **(A)** Cluster A-2 under *Chrysoporthe austroafricana* challenge; **(B)** Cluster A-2 under *Leptocybe invasa* challenge; **(C)** Cluster D-1 under *C. austroafricana* challenge; **(D)** Supercluster C-3, genes within the Ppr1 locus (*Puccinia psidii* resistance gene 1), under *C. austroafricana* challenge. Red: 0–1 (very low–low expression), yellow: 1–10 (low to medium expression), green: 10–220 (medium to very high expression). S_F_C, susceptible, fungal treatment, control; S_F_I, susceptible, fungal treatment, inoculated; R_F_C, resistant, fungal treatment, control; R_F_I, resistant, fungal treatment, inoculated; S_I_C, susceptible, insect treatment, control; S_I_I, susceptible, insect treatment, infested; R_I_C, resistant, insect treatment, control; R_I_I, resistant, insect treatment, infested; N, NB-ARC domain; L, Leucine-rich repeat domain; T, Toll or interleukin-1 receptor domain; C, Coiled-coil domain; noN, no NB-ARC domain.

We identified physical clusters of significant DE for both treatments suggesting that the expression change of co-located genes may have functional relevance (Figure S5). We term these regions expression hotspots. Expression hotspots containing more than one significantly DE gene in *C. austroafricana* treated plants (28 in all) were located on chromosomes one to nine, with a large number of expressed clusters (11) on chromosome five (Table S1). A cluster of five adjacent *CNL* and *NL* genes on chromosome one (cluster A2, see Figure 6A) are significantly differentially expressed under *C. austroafricana* challenge, however a single putative *CN* gene (Eucgr.A02524), within the cluster, is not significantly up or down-regulated, though expression change is evident within heatmaps. Only six clusters of significantly DE genes were found in *L. invasa* treated plants. Interestingly, significant DE was determined for unassigned putative *NBS-LRR* genes suggesting important functional heterozygote allelic variants, for example Eucgr.L01982 (an incomplete putative TIR with log_2_ fold change up in resistant of 2.6) under *C. austroafricana* challenge. Much of the significant expression response by treatment was down-regulation of clusters of putative *NBS-LRR* genes, though notably two single incomplete *NBS-LRR* genes showed opposite regulation in resistant (up) and susceptible (down) genotypes under *C. austroafricana* challenge (Eucgr.H03112 and Eucgr.H03831; Table S1).

Differential expression between resistant and susceptible genotypes was evident for numerous gene models and clusters (Table S1), though change following treatment was not significant. Cluster A2 (Figure 6B) is an example of localized expression variation within a cluster following *L. invasa* challenge and provides an interesting contrast with expression following *C. austroafricana* challenge (Figure 6A). Within cluster D1 (Figure 6C) following *C. austroafricana* challenge and cluster E22 following *L. invasa* challenge, expression levels are higher in resistant plants but not statistically significant by treatment. On closer examination, the *p*-values based on genotype are highly significant for genes within the clusters (D1 and E22, see Table S1). We also identified fold change variation between susceptible and resistant plants where both are up or down-regulated despite no statistical significance by treatment. Here the difference in transcript abundance may be responsible for the observed resistant phenotype for example, an up-regulated TN gene model (Eucgr.E01683), which has a log_2_ fold change in resistant plants of 5.4 compared to 1.4 in susceptible plants.

Expression following *C. austroafricana* challenge within supercluster C3 indicates DE between susceptible and resistant plants (Figure 6D), though only two down-regulated genes (Eucgr.C02640 and C02631) in resistant plants are significant by treatment. The SC-C3 cluster correlates with the *Ppr1* locus for resistance to *P. psidii*.

## Discussion

### A recently expanded TNL class within *Eucayptus grandis*

We identified, within the *E. grandis* genome, 557 putative full-length *NBS-LRR* genes corresponding to the major class of plant resistance genes (Fluhr, 2001) and a further 660 gene models which incorporated the NB-ARC but lacked the LRR domain. Incomplete gene models (270) were also identified, that incorporated one or more domain(s) common to *NBS-LRR* genes, bringing the full gene list to 1487 (Table 1).

Of the identified complete *NBS-LRR* genes, we determined 174 TNL and 133 CNL (a ratio of ~ 6: 4) corresponding to the two major classes. A further 250 NL sequences that lacked CC or TIR domains were also identified, however the NB-ARC domains for these genes fell within the two clades, TNL (50) and CNL (123) indicating evolutionary relatedness. The numbers and proportions of sequences that lack an amino terminal CC or TIR motif nevertheless indicate a likely functional role for these genes, with differential gene expression in both biotic conditions supporting this (Figure 6). Notably genes lacking these motifs are also abundant across other species, except *A. thaliana* (Figure 5). Gene numbers for other woody species show a reversed ratio of *TNL* to *CNL* genes; *P. trichocarpa* 4: 6 (Kohler et al., 2008), *Malus* × *domestica* 4: 6 (Arya et al., 2014) and *V. vinifera* 3: 7 (Argout et al., 2011, Supplementary data). The comparative gene model numbers and proportions in *E. grandis* indicate a more recent *TNL* expansion, predominantly through tandem duplications (Figure 5). Although *A. thaliana* has similar TNL: CNL ratios (~6:4; Meyers et al., 2003), this appears to be uncommon amongst both woody and herbaceous species. The TIR domain is an important component of innate immunity across species through self-association and ligand specific protein-protein interactions (Ve et al., 2014). Evidence for the requirement of TIR heterodimer associations to form functional pathogen recognition complexes have been characterized in *L. usitatissimum* (flax; Ravensdale et al., 2012) and *A. thaliana* (Williams et al., 2014) TNL proteins. Perhaps heterodimerization of *E. grandis* TNL gene products occurs in a similar manner helping to explain the remarkable adaptability of this species.

Our phylogenetic analysis indicated an expected clear evolutionary divergence of the TNL and CNL groups (Figure 3A) as determined for other species (Meyers et al., 2003; Kohler et al., 2008). Clades of highly related sequences, based on phylogenies, show numerous examples of complete and partial *NBS-LRR* genes, which are closely associated physically (on chromosomes). These indicate recent duplications of existing genes, potentially adding variants to the plant resistance gene repertoire. The comparative numbers of full gene models, including genes lacking CC or TIR domains, that aligned with TNL and CNL NB-ARC structures were 47% (224) and 53% (256), respectively, based on pairwise distances of the reduced set.

### Defense-related domains associated with *NBS-LRR* genes in *E. grandis*

Other notable domains often associated with *NBS-LRR* gene models are the BED *zinc* finger (PF02892; after *BE*AF and *D*REF from Drosophila melanogaster peptides) and other DNA binding domains such as MYB and WRKY transcription factors. These domains are largely involved in regulating transcription. We only determined one *NBS-LRR* gene model with the BED fused domain in *E. grandis* (Eucgr.F00886), compared to 34 in *P. trichocarpa* (Kohler et al., 2008; Germain and Séguin, 2011). We also identified a fused MYB-like DNA binding domain (PF00249) within an incomplete *NBS-LRR* gene model (Eucgr.H02157). MYB transcription factors have been found to interact with WRKY transcription factors bound to activated CNL recognition proteins in barley (Chang et al., 2013), thereby initiating defense gene expression. *A. thaliana* has a notable example of a TNL fused to a WRKY domain within Resistance to *Ralstonia solanacearum* gene 1 (RRS1-R; Le Roux et al., 2015). This fused DNA binding domain is believed to act as a decoy target for pathogen secreted molecules attempting to evade basal immune responses (Sarris et al., 2015). Our search, similarly to the *NBS-LRR* scrutiny of the *P. trichocarpa* genome (Kohler et al., 2008), did not identify any WRKY fused domains however the presence of BED and MYB fused domains may indicate similar decoys to RRS1-R. There are 174 MYB and 87 MYB-related transcription factors computationally annotated within the *E. grandis* genome (Jin et al., 2014). We also identified Jacalin-like lectin domains (PF01419), within four incomplete *NBS-LRR* genes (Eucgr.F01690, Eucgr.I02520, Eucgr.I02547, and Eucgr.I02558). These domains, which bind carbohydrates, are involved in innate immunity across a broad range of organisms (Rüdiger and Gabius, 2002). The sharing of this domain with NB-ARC and TIR domains, as determined in this study, suggests a role in pathogen recognition.

### Physical clusters of *NBS-LRR* genes in *E. grandis*

Apart from an ancient lineage-specific whole-genome duplication event around 110 Ma, *E. grandis* has a very high rate of retained tandem duplications (34%; Myburg et al., 2014). These more recent gene expansions are likely to be due to imperfect pairing during meiosis, segmental duplication and gene conversions (Ober, 2010) and are notably evident for large gene families within *E. grandis* (Külheim et al., 2015; Li et al., 2015). Tandem duplications are three to five times more highly represented in *E. grandis* than other species of the rosids (Kersting et al., 2015). Domain expansions, whereby modular protein coding regions are extended, are also more prevalent in tandemly duplicated genes, generally, and stress-related genes, in particular, within *E. grandis* (Kersting et al., 2015). *NBS-LRR* gene regions have also been identified as being over-represented in presence/absence variation of exons in *A. thaliana* likely due to relaxed selection in duplicated genic regions (Bush et al., 2014). To retain duplicated genes over time, they must have functional relevance. However, multiple gene copies also enable mutations to be incorporated leading to functional divergence. The clustering and “birth and death” of *NBS-LRR* genes through evolutionary time has been suggested as a mechanism for highly diversified responses to pathogen evolution (Michelmore and Meyers, 1998).

Most putative *NBS-LRR* genes within *E. grandis* are located within clusters (76%) and in superclusters (71%; Table S1). Only 24% of *E. grandis* putative *NBS-LRR* genes are singletons in comparison to *P. trichocarpa* (37%) and *A. thaliana* (26.8%; (Kohler et al., 2008)). Though clusters of *NBS-LRR* genes have previously been defined in other species (at regions ~ 250 kb; Meyers et al., 2003; Ameline-Torregrosa et al., 2008; Tan and Wu, 2012), superclusters are less well defined. Nevertheless, so called “megaclusters” have been discussed for *A. thaliana*, in which *NBS-LRR* genes are within (20 centiMorgan) regions known as major resistance complexes (Holub, 2001). Within both *Brachypodium distachyon* and *M. truncatula* two large, extended clusters were determined by relaxing the definition of cluster (to a region spanning 430 kb for *B. distachyon* and undefined for *M. truncatula*; Ameline-Torregrosa et al., 2008; Tan and Wu, 2012). The biological significance of these superclusters is yet to be determined but such expansive regions would certainly aid in mispairing during recombination, an important mechanism for increasing genetic diversity (Friedman and Baker, 2007).

Clusters were predominantly classed as either TNL-type or CNL-type with fewer mixed-type clusters. Homogeneous clusters are expected due to the mode of gene expansion through tandem duplications. Heterogeneous clusters however, though rarer than homogeneous ones (Figure 4), are also a feature within *A. thaliana* and are suggested to be the result of segmental duplication (Leister, 2004), though stress-related transposition may also be involved (Lisch, 2013). Clustering may facilitate co-expression of important functionally related genes (Michalak, 2008). Pairs of *NBS-LRR* genes are often required for effective resistance and these genes often occur within clusters (Sinapidou et al., 2004). In rice (Ashikawa et al., 2008) and *A. thaliana* (Williams et al., 2014) tandemly residing *NBS-LRR* genes are co-expressed and form functional heterodimers which can interact with pathogen secreted molecules leading to plant resistance. It is therefore likely that clustering in *E. grandis* facilitates beneficial co-transcription in a similar manner with evidence from differential expression cluster hotspots supporting this assertion, for example Eucgr.D00731 and Eucgr.D00732 (Figure 6C).

While the phylogenetic clustering of *E. grandis* putative *NBS-LRR* genes generally supported recent evolution of tandem duplications, we also found occurrences of highly similar sequences from different chromosomes. Potential mechanisms for such translocations include segmental duplication (Leister, 2004) and the activity of transposable elements (Lisch, 2013). The proposed benefit of maintaining duplicated sequences on different chromosomes is that allele homogenization through recombination and gene conversion, likely for tandemly duplicated sequences, is reduced. Termed “recombinational isolation” (Leister, 2004) these “accidental” translocations, evident within *E. grandis* putative *NBS-LRR* genes, are likely to play an important role in gene evolution.

### Partial *NBS-LRR* genes were differentially expressed under biotic challenge

Co-ordination of resistance gene expression, translation and activation is highly regulated in plants in order to avoid unnecessary cellular damage when no threat is present (Boller and Felix, 2009; Källman et al., 2013). Regulation is therefore likely to be complex and only one aspect of the response can be ascertained from messenger RNA expression at single time-points post inoculation. Nevertheless, expression was identified for 1037 and 1047 complete, partial and incomplete putative *NBS-LRR* genes in the *C. austroafricana* and *L. invasa* studies, respectively. Of these, 423 putative complete *NBS-LRR* genes were present within both experiments. Of interest, we noted differential regulation of many chimeric variant putative genes including, but not limited to; Eucgr.B02976 (CNNL), Eucgr.B01237 (CNNL), Eucgr.A01678 (NNL), Eucgr.C02641(TNNNL; Table S1).

The differential lack of putative gene transcripts from resistant and susceptible plants is interesting and may suggest that these alleles are absent in these individuals. Significant differential expression under challenge was evident for complete, partial and incomplete genes indicating that they are important biologically or that they are the result of residual expression due to physical proximity to important functional genes. Expression of incomplete putative *NBS-LRR* genes has been found in *M. esculenta* (cassava; Lozano et al., 2015) and these are suggested to be relics from incomplete tandem gene expansions. The cassava study, in contrast to our study, found only a single partial putative *NBS-LRR* gene differentially expressed in response to Cassava Bacterial Blight.

Though genes within clusters are physically close, on chromosomes, not all genes within expressed clusters show significant DE (Figure 6) suggesting that certain genes do indeed have functional relevance and that these are not merely transcriptionally active zones. Interestingly much of the significant expression response identified was down-regulation of clusters of putative *NBS-LRR* genes, though notably two single incomplete *NBS-LRR* genes showed opposite regulation in resistant (up) and susceptible (down) genotypes under *C. austroafricana* challenge (Eucgr.H03112 and Eucgr.H03831). Effector suppression of resistance gene expression may be occurring as identified in other plant pathogen interactions (Houterman et al., 2008). A further interesting possibility is that the up-regulation and translation of partial genes may be involved in regulatory interactions by blocking functional heterodimers. The interaction of single domain microproteins dimerizing with a protein target can have a dominant negative regulatory effect as determined in transcription factor regulation in plants (Eguen et al., 2015).

### Differential expression of physically clustered genes suggest functional relevance

Comparison of resistant and susceptible genotypes (Figure 6; Table S1), suggests that genotype variation is worth investigating further, in particular for certain loci which harbor numerous DE putative *NBS-LRR* genes, such as the A2 and D1 clusters. Fold change variation in putative *NBS-LRR* genes between susceptible and resistant plants was also observed, where both are up or down-regulated, despite lack of statistical significance by treatment. These variations may account for the observed resistant phenotype due to differential log_2_ fold change. While no *NBS-LRR* genes have yet been cloned for *E. grandis*, the clustered expression patterns indicate that co-located genes, for example cluster D1, may have functional significance, as similarly determined for rice (Ashikawa et al., 2008), flax (Ravensdale et al., 2012) and *A. thaliana* (Saucet et al., 2015) where important receptor pairs form functional dimers.

## Conclusion

Our scrutiny of the *E. grandis* genome for putative *NBS-LRR* genes has identified an extensive gene family with some differences to other woody plant species. Notably the *TNL* genes have recently expanded and *E. grandis* has a higher ratio of TNL to CNL compared to other woody plants. Retention of *NBS-LRR* genes in clusters and superclusters is an important feature for all plants, however *E. grandis* maintains a smaller percentage of single genes compared to *P. trichocarpa* and *A. thaliana*. Both these features are likely to be the result of recent tandem duplications, however selective pressure to retain higher numbers of *TNL*-like genes, occurring in their genomic arrangements, must play an important role. We found that a large proportion of putative *NBS-LRR* genes are expressed, including 423 complete gene models, and are therefore transcriptionally active. We further identified clusters where several putative complete and partial *NBS-LRR* genes were differentially expressed under challenge and this was specific to the type of challenge (fungal or insect). The clustering of putative *NBS-LRR* genes correlates with differential expression responses for many clusters suggesting functional relevance for the physical arrangement of this gene family. The findings of this study therefore provide researchers with a resource to investigate specificity of host response to biotic stress in an important forest species.

## Author contributions

NC and PT shared the lead author duties for this manuscript. NC carried out much of the data analysis and aspects of the manuscript drafting. PT conducted early data and gene expression analysis and much of the manuscript writing. SN and CK were involved in the design and co-ordination of the study and conducted some of the expression analysis and writing. All authors read and approved the final manuscript.

## Funding

Top up scholarships were generously provided for PT from the University of Sydney and Rural Industries Research and Development Corporation, Australia.

### Conflict of interest statement

The authors declare that the research was conducted in the absence of any commercial or financial relationships that could be construed as a potential conflict of interest.

## Supplementary material

Table S1 **Full list of *Eucalyptus grandis* putative NBS-LRR genes sorted by position on the genome**. Information per gene includes the chromosomal position, class, physical cluster and phylogeny clade membership, identification method, raw expression data, log_2_ fold change values and ANOVA results (*p*-values). S_F_C, susceptible, fungal treatment, control; S_F_I, susceptible, fungal treatment, inoculated; R_F_C, resistant, fungal treatment, control; R_F_I, resistant, fungal treatment, inoculated; S_I_C, susceptible, insect treatment, control; S_I_I, susceptible, insect treatment, infested; R_I_C, resistant, insect treatment, control; R_I_I, resistant, insect treatment, infested.

Table S2 **Conserved amino acid sequences for NB-ARC and TIR motifs from MEME analysis with CNL-like and TNL-like gene models in *Eucalyptus grandis* (*Eg*) and *Arabidopsis thaliana* (*At*; Meyers et al., 2003)**. The expected amino acid tryptophan (W) is identified in the Kinase 2 subdomain for CNL sequences–underlined.

Figure S1 **Neighbor joining tree of 480 *Eucalyptus grandis* NB-ARC domains from complete NBS-LRR genes**. The tree is drawn to scale, with branch lengths in the same units as those of the evolutionary distances used to infer the phylogenetic tree. The evolutionary distances were computed using the p-distance method and are in the units of the number of amino acid differences per site. The analysis involved 495 amino acid sequences (480 *E. grandis*). All ambiguous positions were removed for each sequence pair.

Figure S2 **Neighbor joining tree of 616 *Eucalyptus grandis* NB-ARC domains from all non-TIR NBS-LRR-like genes**. The tree is drawn to scale, with branch lengths in the same units as those of the evolutionary distances used to infer the phylogenetic tree. The evolutionary distances were computed using the p-distance method and are in the units of the number of amino acid differences per site. The analysis involved 631 amino acid sequences (616 *E. grandis*). All ambiguous positions were removed for each sequence pair.

Figure S3 **Neighbor joining tree of 396 *Eucalyptus grandis* NB-ARC domains from all TIR NBS-LRR-like genes**. The tree is drawn to scale, with branch lengths in the same units as those of the evolutionary distances used to infer the phylogenetic tree. The evolutionary distances were computed using the p-distance method and are in the units of the number of amino acid differences per site. The analysis involved 411 amino acid sequences (396 *E. grandis*). All ambiguous positions were removed for each sequence pair.

Figure S4 **The definition of a (A) cluster and a (B) supercluster is illustrated using a region (starting at 13 Mb and ending at 18 Mb) on chromosome 4**.

Figure S5 **Physical locations for all complete, partial, and incomplete *NBS-LRR* gene models that were expressed under challenge of *Chrysoporthe austroafricana* and *Leptocybe invasa* on *Eucalyptus grandis* chromosomes (Mapchart)**. Variation in means from treatment (ANOVA) were identified based on significance ^*^*p* < 0.01, ^**^*p* < 0.001, ^***^*p* < 0.0001 (^***^ are also underlined) and log_2_ gene expression ratios greater than 1 or smaller than −1 for resistant and susceptible plants. Color distinguishes between different classes (TNL = pink, CNL = green, NL = red, incomplete NL = black, BLAST homolog non-NL = black). Scale bar = Mb. Cluster and supercluster regions are indicated and *E. grandis* gene IDs are provided.

Figure S6 **NB-ARC-LRR fused domains (A) and TIR-NB-ARC-LRR fused domains (B)**. Conserved amino acid sequences are indicated with lines (top). The GKT (Kinase 1) conserved motif is recognized as a P-loop structure important in ATP hydrolysis while the hDD is also well conserved in NB-ARC domains (Kinase 2) as important in co-ordinating Mg^2+^ as a co-factor (Tameling et al., 2006). These two important sub-domains of NB-ARC are sometimes termed the Walker A and Walker B motifs (Walker et al., 1982) and are identified as A and B, respectively, within the I-Tasser protein structures (bottom) for a representative CNL (Eucgr.L01363) and TNL (Eucgr.C00020) sequence from the *Eucalyptus grandis* genome.
