# Supplementary figures and images for: The Eucalyptus grandis NBS-LRR Gene Family: Physical Clustering and Expression Hotspots
Source: Front Plant Sci. 2016 Jan 12;6:1238. doi: 10.3389/fpls.2015.01238 (PMC4709456; doi:10.3389/fpls.2015.01238)

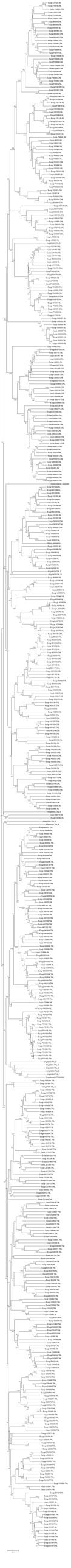

Supplement: Figure S1 — Neighbor joining tree of 480 Eucalyptus grandis NB-ARC domains from complete NBS-LRR genes. The tree is drawn to scale, with branch lengths in the same units as those of the evolutionary distances used to infer the phylogenetic tree. The evolutionary distances were computed using the p-distance method and are in the units of the number of amino acid differences per site. The analysis involved 495 amino acid sequences (480 E. grandis). All ambiguous positions were removed for each sequence pair. [file Image1.PDF]

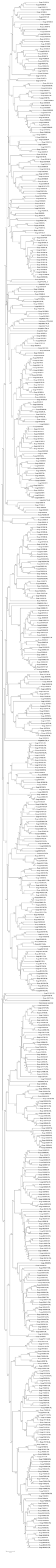

Supplement: Figure S2 — Neighbor joining tree of 616 Eucalyptus grandis NB-ARC domains from all non-TIR NBS-LRR-like genes. The tree is drawn to scale, with branch lengths in the same units as those of the evolutionary distances used to infer the phylogenetic tree. The evolutionary distances were computed using the p-distance method and are in the units of the number of amino acid differences per site. The analysis involved 631 amino acid sequences (616 E. grandis). All ambiguous positions were removed for each sequence pair. [file Image2.PDF]

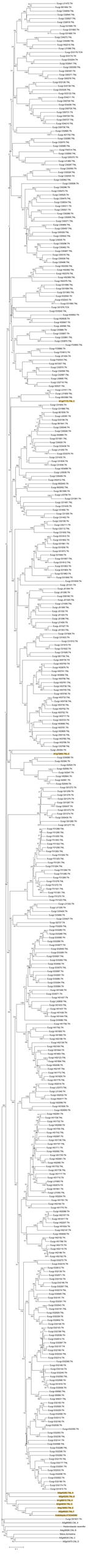

Supplement: Figure S3 — Neighbor joining tree of 396 Eucalyptus grandis NB-ARC domains from all TIR NBS-LRR-like genes. The tree is drawn to scale, with branch lengths in the same units as those of the evolutionary distances used to infer the phylogenetic tree. The evolutionary distances were computed using the p-distance method and are in the units of the number of amino acid differences per site. The analysis involved 411 amino acid sequences (396 E. grandis). All ambiguous positions were removed for each sequence pair. [file Image3.PDF]
