# Supplementary material for: The Eucalyptus grandis NBS-LRR Gene Family: Physical Clustering and Expression Hotspots
Source: Front Plant Sci. 2016 Jan 12;6:1238. doi: 10.3389/fpls.2015.01238 (PMC4709456; doi:10.3389/fpls.2015.01238)
Supplement: Figure S4 — The definition of a (A) cluster and a (B) supercluster is illustrated using a region (starting at 13 Mb and ending at 18 Mb) on chromosome 4. [file Image4.PDF]

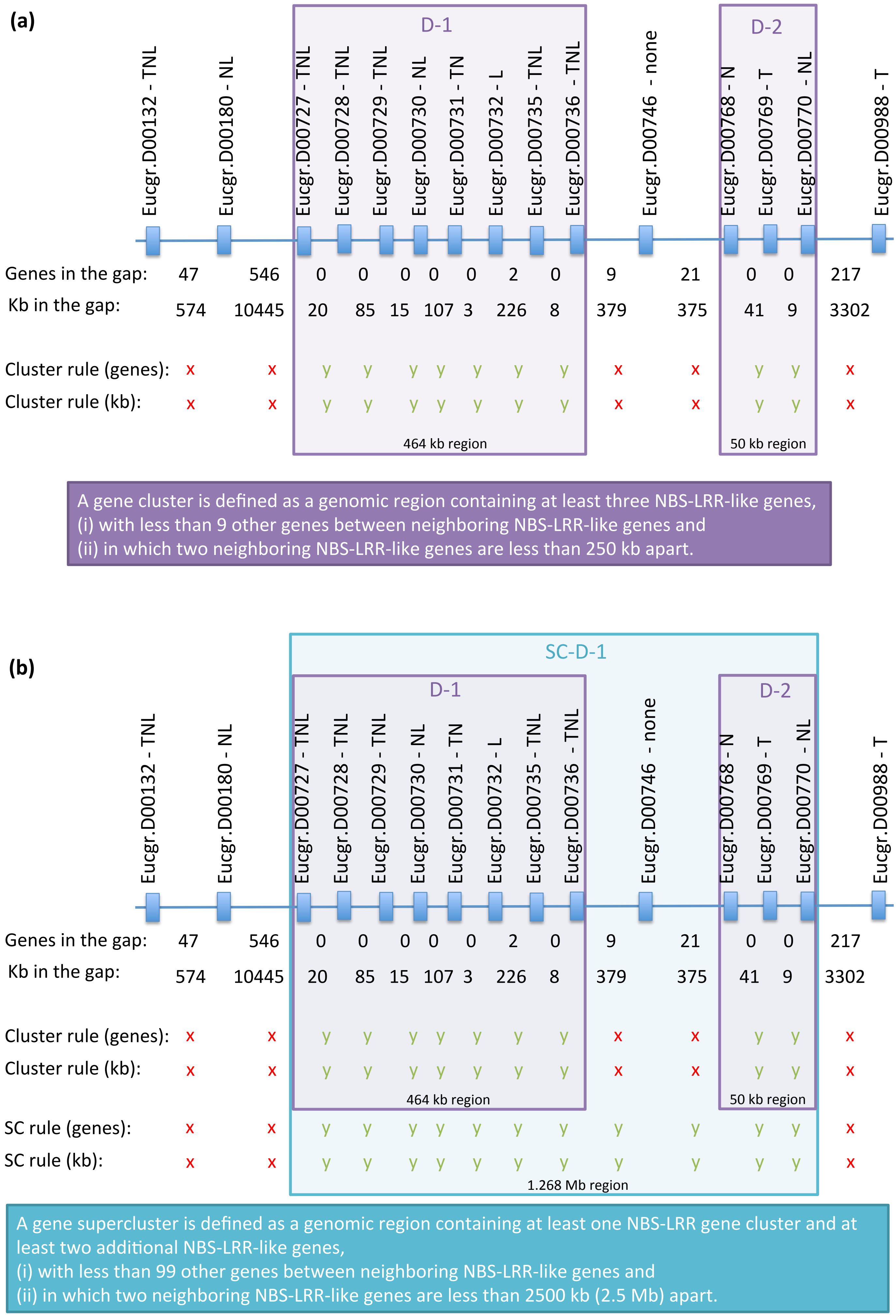

(a)
D-1
D-2
Eucgr.D00132 - TNL
Eucgr.D00180 - NL
Eucgr.D00727 - TNL
Eucgr.D00728 - TNL
Eucgr.D00729 - TNL
Eucgr.D00730 - NL
Eucgr.D00731 - TN
Eucgr.D00732 - L
Eucgr.D00735 - TNL
Eucgr.D00736 - TNL
Eucgr.D00746 - none
Eucgr.D00768 - N
Eucgr.D00769 - T
Eucgr.D00770 - NL
Eucgr.D00988 - T
Genes in the gap: 47 546 0 0 0 0 0 2 0 9 21 0 0 217
Kb in the gap: 574 10445 20 85 15 107 3 226 8 379 375 41 9 3302
Cluster rule (genes): x x y y y y y y y x x y y x
Cluster rule (kb): x x y y y y y y y x x y y x
464 kb region
50 kb region
A gene cluster is defined as a genomic region containing at least three NBS-LRR-like genes,
(i) with less than 9 other genes between neighboring NBS-LRR-like genes and
(ii) in which two neighboring NBS-LRR-like genes are less than 250 kb apart.
(b)
SC-D-1
D-1
D-2
Eucgr.D00132 - TNL
Eucgr.D00180 - NL
Eucgr.D00727 - TNL
Eucgr.D00728 - TNL
Eucgr.D00729 - TNL
Eucgr.D00730 - NL
Eucgr.D00731 - TN
Eucgr.D00732 - L
Eucgr.D00735 - TNL
Eucgr.D00736 - TNL
Eucgr.D00746 - none
Eucgr.D00768 - N
Eucgr.D00769 - T
Eucgr.D00770 - NL
Eucgr.D00988 - T
Genes in the gap: 47 546 0 0 0 0 0 2 0 9 21 0 0 217
Kb in the gap: 574 10445 20 85 15 107 3 226 8 379 375 41 9 3302
Cluster rule (genes): x x y y y y y y y x x y y x
Cluster rule (kb): x x y y y y y y y x x y y x
464 kb region
50 kb region
SC rule (genes): x x y y y y y y y y y y y x
SC rule (kb): x x y y y y y y y y y y y x
1.268 Mb region
A gene supercluster is defined as a genomic region containing at least one NBS-LRR gene cluster and at least two additional NBS-LRR-like genes,
(i) with less than 99 other genes between neighboring NBS-LRR-like genes and
(ii) in which two neighboring NBS-LRR-like genes are less than 2500 kb (2.5 Mb) apart.
