# Supplementary material for: The Eucalyptus grandis NBS-LRR Gene Family: Physical Clustering and Expression Hotspots
Source: Front Plant Sci. 2016 Jan 12;6:1238. doi: 10.3389/fpls.2015.01238 (PMC4709456; doi:10.3389/fpls.2015.01238)
Supplement: Figure S5 — Physical locations for all complete, partial, and incomplete NBS-LRR gene models that were expressed under challenge of Chrysoporthe austroafricana and Leptocybe invasa on Eucalyptus grandis chromosomes (Mapchart). Variation in means from treatment (ANOVA) were identified based on significance *p < 0.01, **p < 0.001, ***p < 0.0001 (*** are also underlined) and log2 gene expression ratios greater than 1 or smaller than −1 for resistant and susceptible plants. Color distinguishes between different classes (TNL = pink, CNL = green, NL = red, incomplete NL = black, BLAST homolog non-NL = black). Scale bar = Mb. Cluster and supercluster regions are indicated and E. grandis gene IDs are provided. [file Image5.pdf]

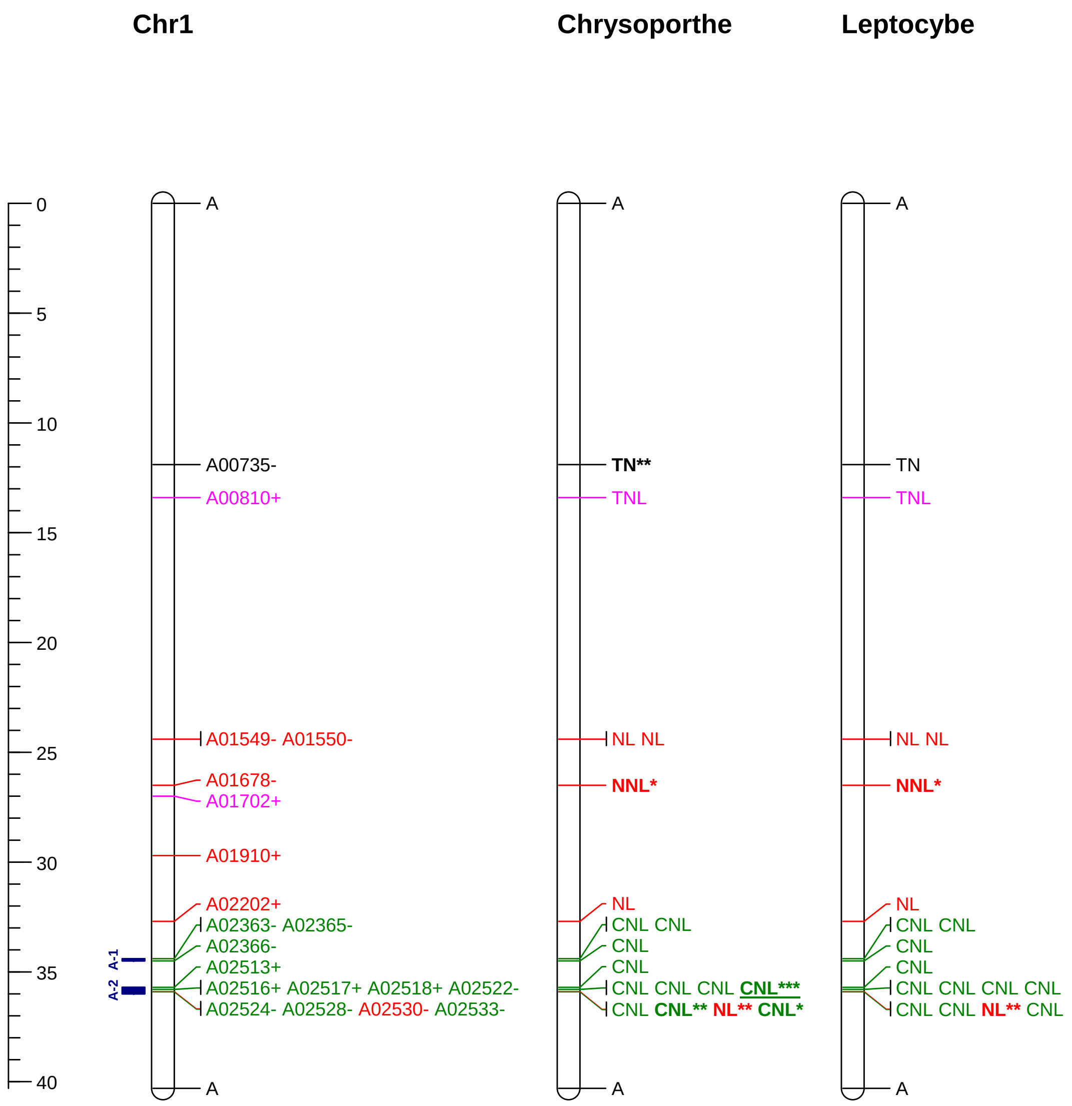
Chr1
Chrysoporthe
Leptocybe
0
5
10
15
20
25
30
35
40
A-1
A-2
A
A00735-
A00810+
A01549- A01550-
A01678-
A01702+
A01910+
A02202+
A02363- A02365-
A02366-
A02513+
A02516+ A02517+ A02518+ A02522-
A02524- A02528- A02530- A02533-
A
A
TN**
TNL
NL NL
NNL*
NL
CNL CNL
CNL
CNL
CNL CNL CNL CNL***
CNL CNL** NL** CNL*
A
A
TN
TNL
NL NL
NNL*
NL
CNL CNL
CNL
CNL
CNL CNL CNL CNL
CNL CNL NL** CNL
A

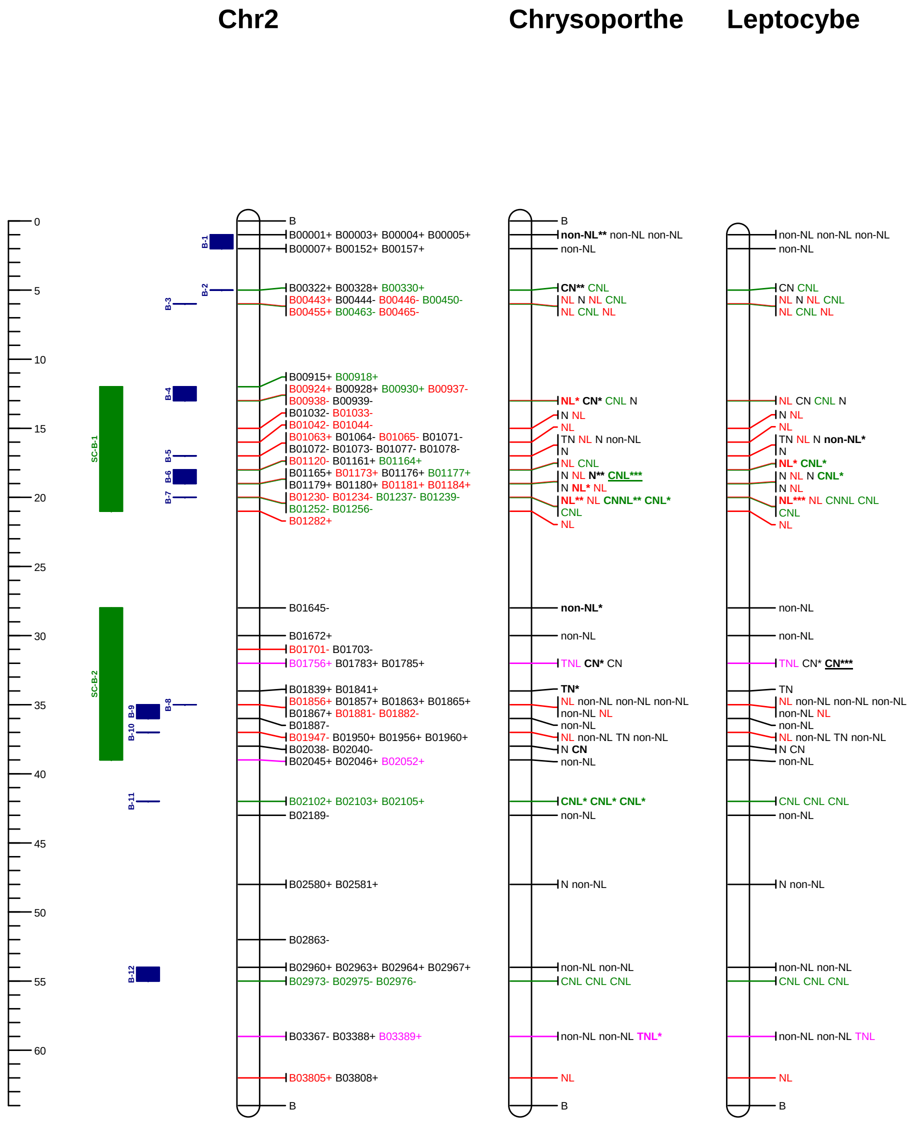

Chr2
Chrysoporthe
Leptocybe
0
5
10
15
20
25
30
35
40
45
50
55
60
SC-B-1
SC-B-2
B-1
B-2
B-3
B-4
B-5
B-6
B-7
B-8
B-9
B-10
B-11
B-12
B
B00001+ B00003+ B00004+ B00005+
B00007+ B00152+ B00157+
B00322+ B00328+ B00330+
B00443+ B00444- B00446- B00450-
B00455+ B00463- B00465-
B00915+ B00918+
B00924+ B00928+ B00930+ B00937-
B00938- B00939-
B01032- B01033-
B01042- B01044-
B01063+ B01064- B01065- B01071-
B01072- B01073- B01077- B01078-
B01120- B01161+ B01164+
B01165+ B01173+ B01176+ B01177+
B01179+ B01180+ B01181+ B01184+
B01230- B01234- B01237- B01239-
B01252- B01256-
B01282+
B01645-
B01672+
B01701- B01703-
B01756+ B01783+ B01785+
B01839+ B01841+
B01856+ B01857+ B01863+ B01865+
B01867+ B01881- B01882-
B01887-
B01947- B01950+ B01956+ B01960+
B02038- B02040-
B02045+ B02046+ B02052+
B02102+ B02103+ B02105+
B02189-
B02580+ B02581+
B02863-
B02960+ B02963+ B02964+ B02967+
B02973- B02975- B02976-
B03367- B03388+ B03389+
B03805+ B03808+
B
B
non-NL** non-NL non-NL
non-NL
CN** CNL
NL N NL CNL
NL CNL NL
NL* CN* CNL N
N NL
NL
TN NL N non-NL
N
NL CNL
N NL N** CNL***
N NL* NL
NL** NL CNNL** CNL*
CNL
NL
non-NL*
non-NL
TNL CN* CN
TN*
NL non-NL non-NL non-NL
non-NL NL
non-NL
NL non-NL TN non-NL
N CN
non-NL
CNL* CNL* CNL*
non-NL
N non-NL
non-NL non-NL
CNL CNL CNL
non-NL non-NL TNL*
NL
B
non-NL non-NL non-NL
non-NL
CN CNL
NL N NL CNL
NL CNL NL
NL CN CNL N
N NL
NL
TN NL N non-NL*
N
NL* CNL*
N NL N CNL*
N NL
NL*** NL CNNL CNL
CNL
NL
non-NL
non-NL
TNL CN* CN***
TN
NL non-NL non-NL non-NL
non-NL NL
non-NL
NL non-NL TN non-NL
N CN
non-NL
CNL CNL CNL
non-NL
N non-NL
non-NL non-NL
CNL CNL CNL
non-NL non-NL TNL
NL
B

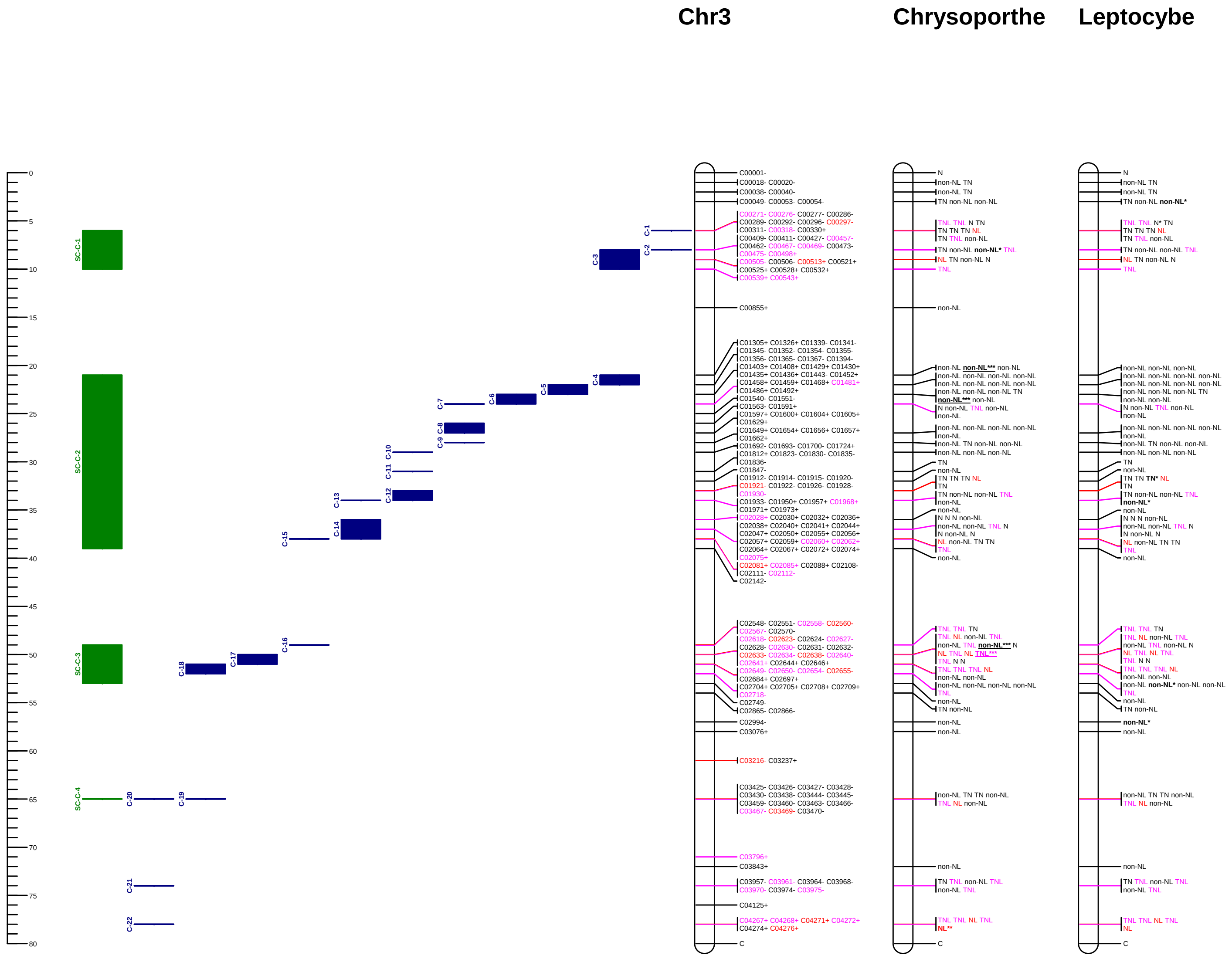

Chr3
Chrysoporthe
Leptocybe

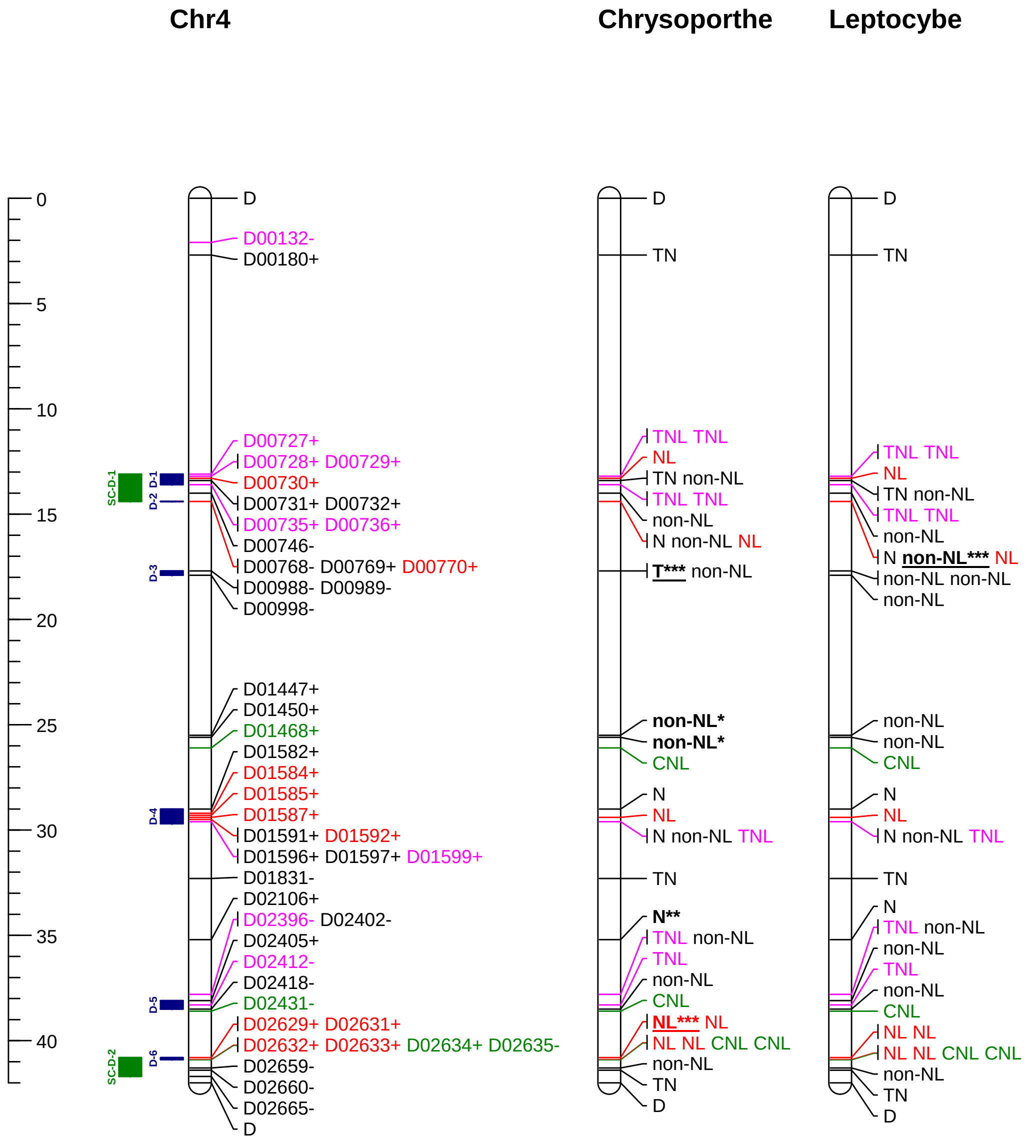

Chr4
Chrysoporthe
Leptocybe
0
5
10
15
20
25
30
35
40
SC-D-1
SC-D-2
D-1
D-2
D-3
D-4
D-5
D-6
D
D00132-
D00180+
D00727+
D00728+ D00729+
D00730+
D00731+ D00732+
D00735+ D00736+
D00746-
D00768- D00769+ D00770+
D00988- D00989-
D00998-
D01447+
D01450+
D01468+
D01582+
D01584+
D01585+
D01587+
D01591+ D01592+
D01596+ D01597+ D01599+
D01831-
D02106+
D02396- D02402-
D02405+
D02412-
D02418-
D02431-
D02629+ D02631+
D02632+ D02633+ D02634+ D02635-
D02659-
D02660-
D02665-
D
D
TN
TNL TNL
NL
TN non-NL
TNL TNL
non-NL
N non-NL NL
T*** non-NL
non-NL*
non-NL*
CNL
N
NL
N non-NL TNL
TN
N**
TNL non-NL
TNL
non-NL
CNL
NL*** NL
NL NL CNL CNL
non-NL
TN
D
D
TN
TNL TNL
NL
TN non-NL
TNL TNL
non-NL
N non-NL*** NL
non-NL non-NL
non-NL
non-NL
non-NL
CNL
N
NL
N non-NL TNL
TN
N
TNL non-NL
non-NL
TNL
non-NL
CNL
NL NL
NL NL CNL CNL
non-NL
TN
D

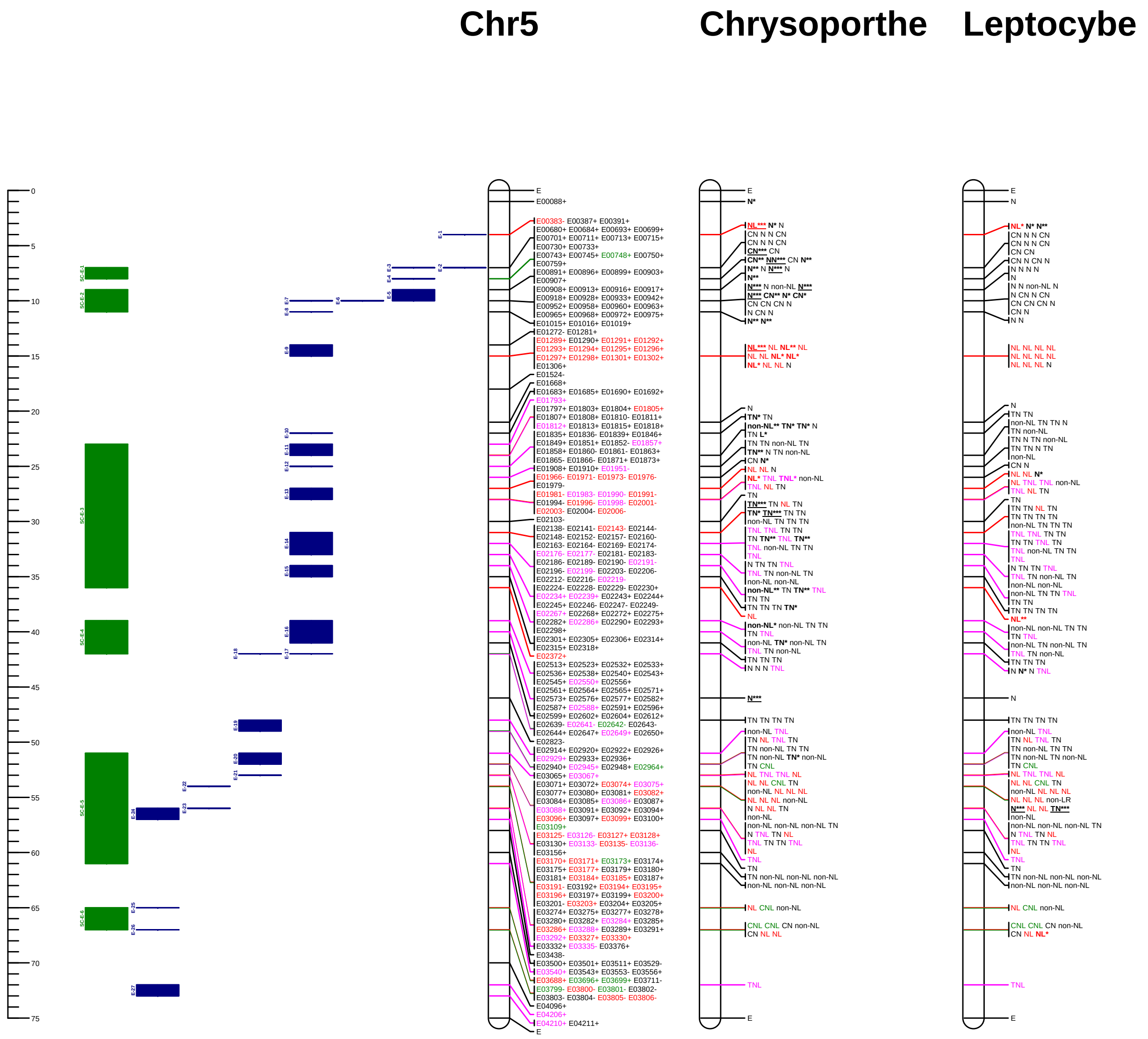

Chr5
Chrysoporthe
Leptocybe
0
5
10
15
20
25
30
35
40
45
50
55
60
65
70
75

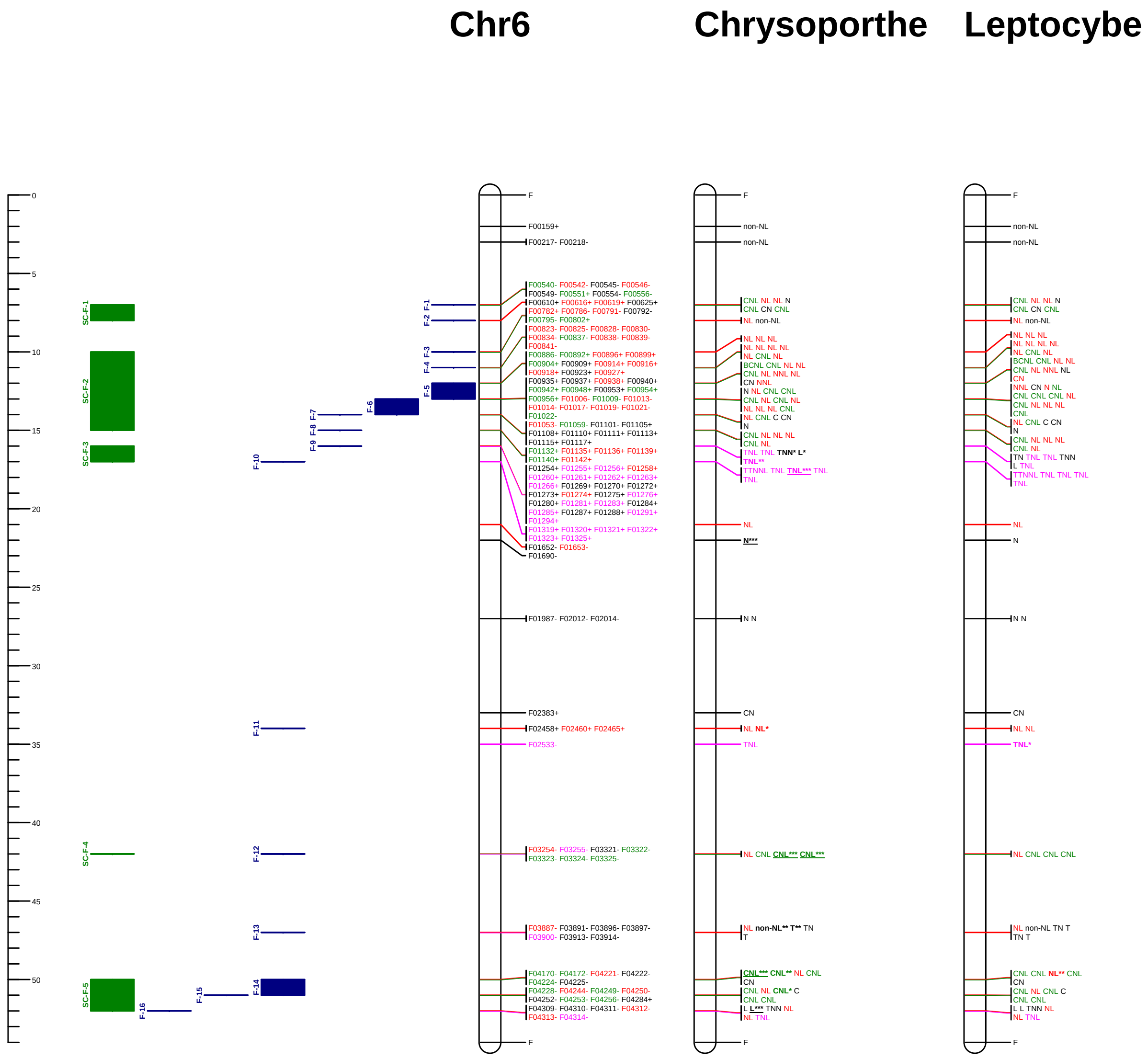

Chr6
Chrysoporthe
Leptocybe
0
5
10
15
20
25
30
35
40
45
50
SC-F-1
SC-F-2
SC-F-3
SC-F-4
SC-F-5
F-1
F-2
F-3
F-4
F-5
F-6
F-7
F-8
F-9
F-10
F-11
F-12
F-13
F-14
F-15
F-16
F
F00159+
F00217- F00218-
F00540- F00542- F00545- F00546-
F00549- F00551+ F00554- F00556-
F00610+ F00616+ F00619+ F00625+
F00782+ F00786- F00791- F00792-
F00795- F00802+
F00823- F00825- F00828- F00830-
F00834- F00837- F00838- F00839-
F00841-
F00886- F00892+ F00896+ F00899+
F00904+ F00909+ F00914+ F00916+
F00918+ F00923+ F00927+
F00935+ F00937+ F00938+ F00940+
F00942+ F00948+ F00953+ F00954+
F00956+ F01006- F01009- F01013-
F01014- F01017- F01019- F01021-
F01022-
F01053- F01059- F01101- F01105+
F01108+ F01110+ F01111+ F01113+
F01115+ F01117+
F01132+ F01135+ F01136+ F01139+
F01140+ F01142+
F01254+ F01255+ F01256+ F01258+
F01260+ F01261+ F01262+ F01263+
F01266+ F01269+ F01270+ F01272+
F01273+ F01274+ F01275+ F01276+
F01280+ F01281+ F01283+ F01284+
F01285+ F01287+ F01288+ F01291+
F01294+
F01319+ F01320+ F01321+ F01322+
F01323+ F01325+
F01652- F01653-
F01690-
F01987- F02012- F02014-
F02383+
F02458+ F02460+ F02465+
F02533-
F03254- F03255- F03321- F03322-
F03323- F03324- F03325-
F03887- F03891- F03896- F03897-
F03900- F03913- F03914-
F04170- F04172- F04221- F04222-
F04224- F04225-
F04228- F04244- F04249- F04250-
F04252- F04253- F04256- F04284+
F04309- F04310- F04311- F04312-
F04313- F04314-
F
F
non-NL
non-NL
CNL NL NL N
CNL CN CNL
NL non-NL
NL NL NL
NL NL NL NL
NL CNL NL
BCNL CNL NL NL
CNL NL NNL NL
CN NNL
N NL CNL CNL
CNL NL CNL NL
NL NL NL CNL
NL CNL C CN
N
CNL NL NL NL
CNL NL
TNL TNL TNN* L*
TNL**
TTNNL TNL TNL*** TNL
TNL
NL
N***
N N
CN
NL NL*
TNL
NL CNL CNL*** CNL***
NL non-NL** T** TN
T
CNL*** CNL** NL CNL
CN
CNL NL CNL* C
CNL CNL
L L*** TNN NL
NL TNL
F
F
non-NL
non-NL
CNL NL NL N
CNL CN CNL
NL non-NL
NL NL NL
NL NL NL NL
NL CNL NL
BCNL CNL NL NL
CNL NL NNL NL
CN
NNL CN N NL
CNL CNL CNL NL
CNL NL NL NL
CNL
NL CNL C CN
N
CNL NL NL NL
CNL NL
TN TNL TNL TNN
L TNL
TTNNL TNL TNL TNL
TNL
NL
N
N N
CN
NL NL
TNL*
NL CNL CNL CNL
NL non-NL TN T
TN T
CNL CNL NL** CNL
CN
CNL NL CNL C
CNL CNL
L L TNN NL
NL TNL
F

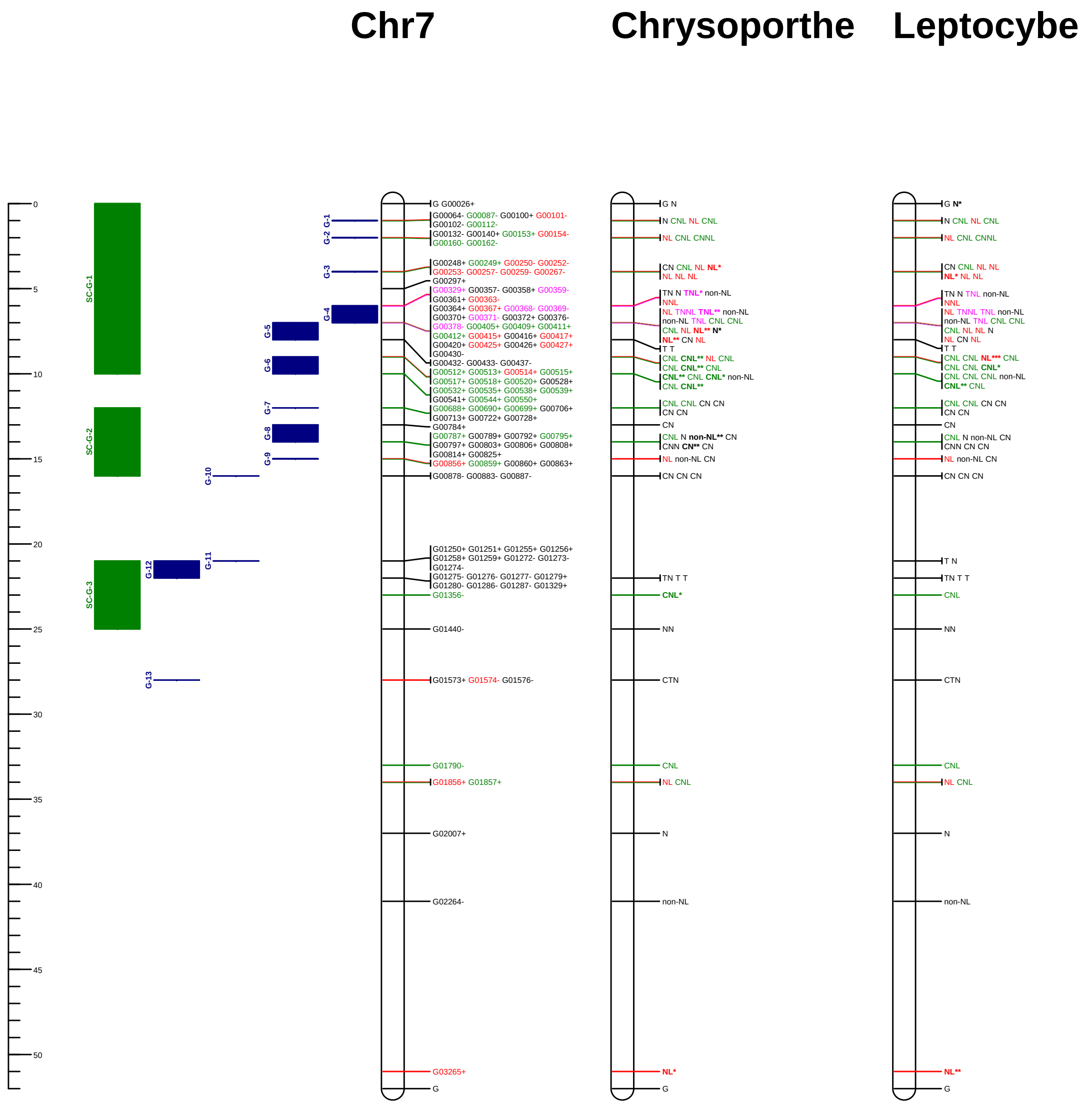
Chr7
Chrysoporthe
Leptocybe
SC-G-1
SC-G-2
SC-G-3
G-1
G-2
G-3
G-4
G-5
G-6
G-7
G-8
G-9
G-10
G-11
G-12
G-13

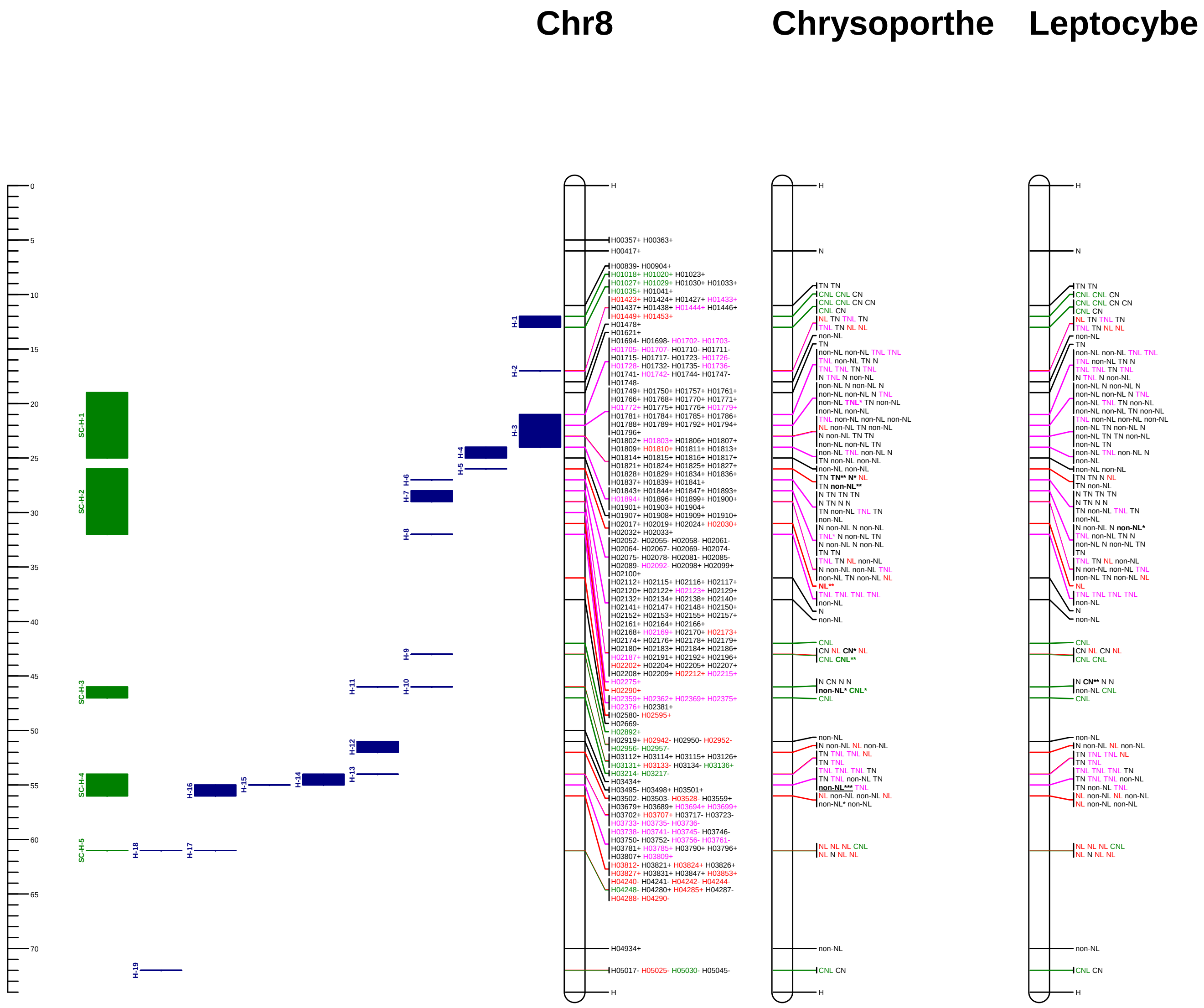
Chr8
Chrysoporthe
Leptocybe
0
5
10
15
20
25
30
35
40
45
50
55
60
65
70
SC-H-1
SC-H-2
SC-H-3
SC-H-4
SC-H-5
H-1
H-2
H-3
H-4
H-5
H-6
H-7
H-8
H-9
H-10
H-11
H-12
H-13
H-14
H-15
H-16
H-17
H-18
H-19

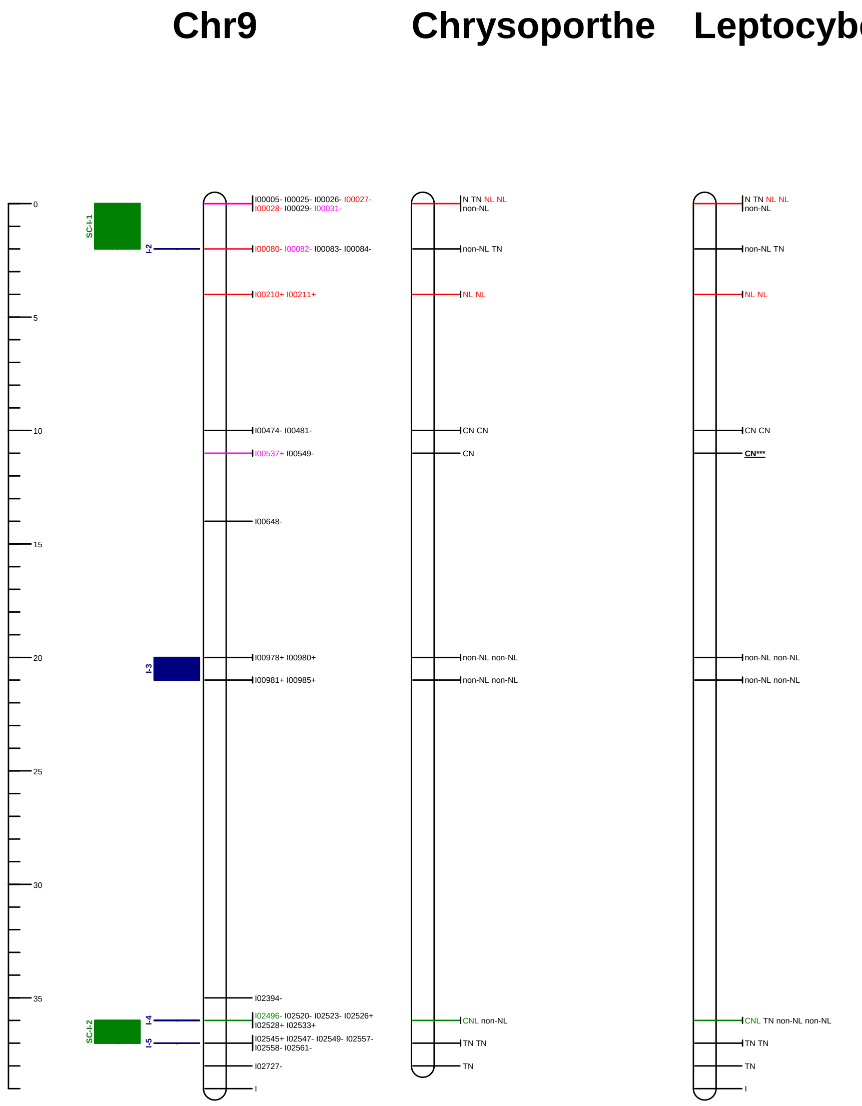
Chr9
Chrysoporthe
Leptocybe
0
5
10
15
20
25
30
35
SC-I-1
I-2
I-3
SC-I-2
I-4
I-5
I00005- I00025- I00026- I00027-
I00028- I00029- I00031-
I00080- I00082- I00083- I00084-
I00210+ I00211+
I00474- I00481-
I00537+ I00549-
I00648-
I00978+ I00980+
I00981+ I00985+
I02394-
I02496- I02520- I02523- I02526+
I02528+ I02533+
I02545+ I02547- I02549- I02557-
I02558- I02561-
I02727-
I
N TN NL NL
non-NL
non-NL TN
NL NL
CN CN
CN
non-NL non-NL
non-NL non-NL
CNL non-NL
TN TN
TN
N TN NL NL
non-NL
non-NL TN
NL NL
CN CN
CN***
non-NL non-NL
non-NL non-NL
CNL TN non-NL non-NL
TN TN
TN
I

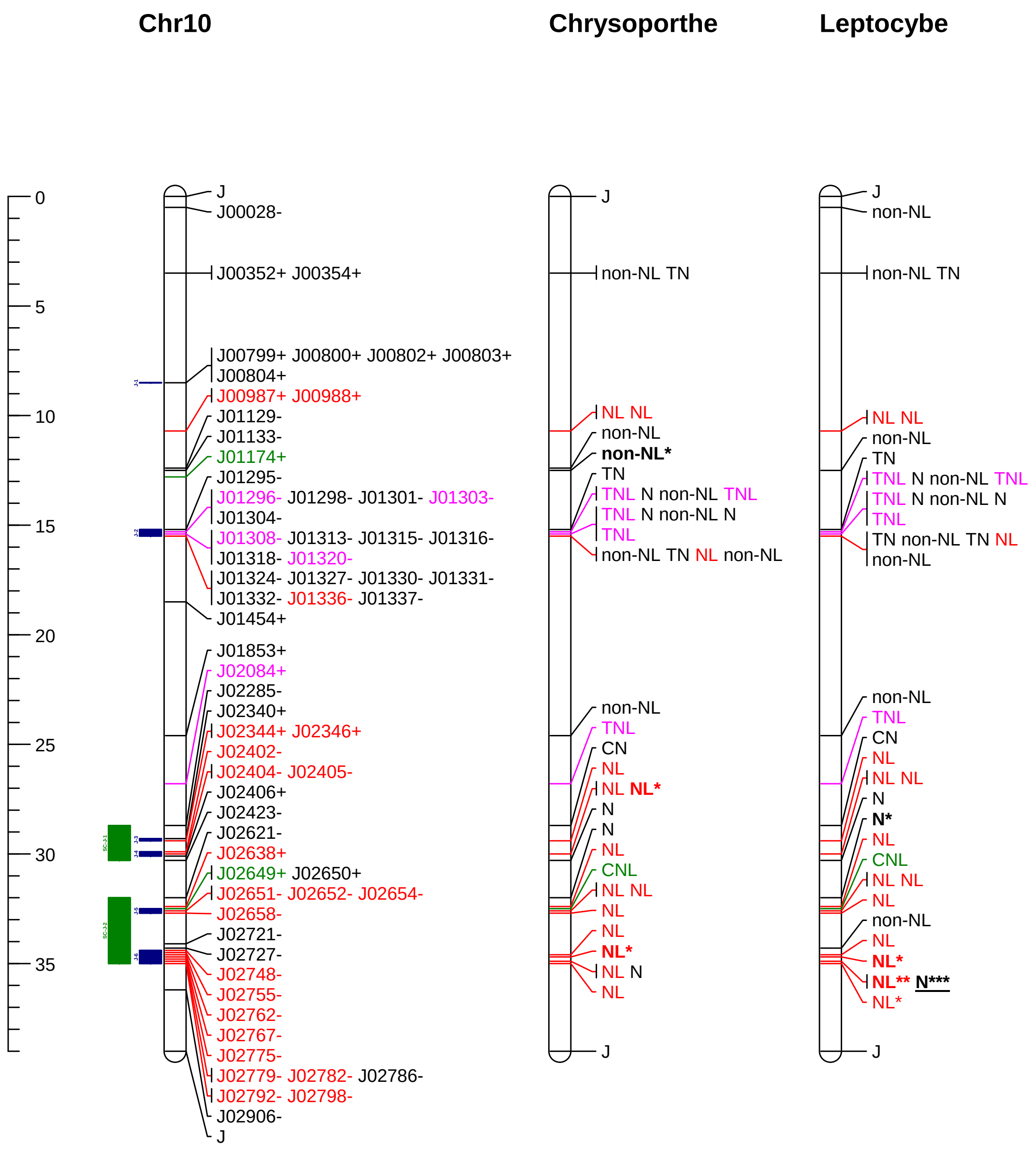

Chr10
Chrysoporthe
Leptocybe
0
5
10
15
20
25
30
35
J
J00028-
J00352+ J00354+
J00799+ J00800+ J00802+ J00803+
J00804+
J00987+ J00988+
J01129-
J01133-
J01174+
J01295-
J01296- J01298- J01301- J01303-
J01304-
J01308- J01313- J01315- J01316-
J01318- J01320-
J01324- J01327- J01330- J01331-
J01332- J01336- J01337-
J01454+
J01853+
J02084+
J02285-
J02340+
J02344+ J02346+
J02402-
J02404- J02405-
J02406+
J02423-
J02621-
J02638+
J02649+ J02650+
J02651- J02652- J02654-
J02658-
J02721-
J02727-
J02748-
J02755-
J02762-
J02767-
J02775-
J02779- J02782- J02786-
J02792- J02798-
J02906-
J
J
non-NL TN
NL NL
non-NL
non-NL*
TN
TNL N non-NL TNL
TNL N non-NL N
TNL
non-NL TN NL non-NL
non-NL
TNL
CN
NL
NL NL*
N
N
NL
CNL
NL NL
NL
NL
NL*
NL N
NL
J
J
non-NL
non-NL TN
NL NL
non-NL
TN
TNL N non-NL TNL
TNL N non-NL N
TNL
TN non-NL TN NL
non-NL
non-NL
TNL
CN
NL
NL NL
N
N*
NL
CNL
NL NL
NL
non-NL
NL
NL*
NL** N***
NL*
J

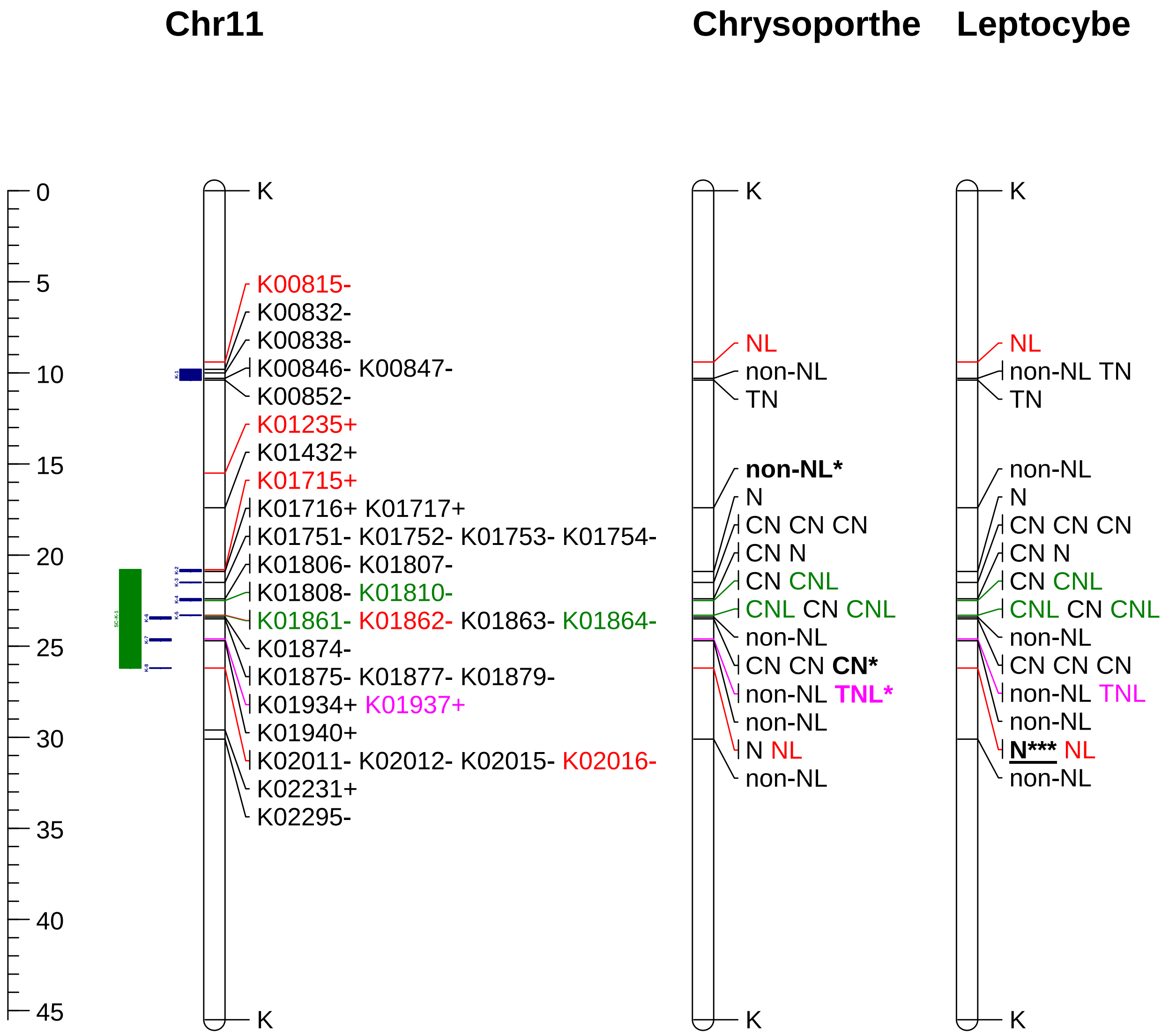

Chr11
Chrysoporthe
Leptocybe
0
5
10
15
20
25
30
35
40
45
K
K00815-
K00832-
K00838-
K00846- K00847-
K00852-
K01235+
K01432+
K01715+
K01716+ K01717+
K01751- K01752- K01753- K01754-
K01806- K01807-
K01808- K01810-
K01861- K01862- K01863- K01864-
K01874-
K01875- K01877- K01879-
K01934+ K01937+
K01940+
K02011- K02012- K02015- K02016-
K02231+
K02295-
K
K
NL
non-NL
TN
non-NL*
N
CN CN CN
CN N
CN CNL
CNL CN CNL
non-NL
CN CN CN*
non-NL TNL*
non-NL
N NL
non-NL
K
K
NL
non-NL TN
TN
non-NL
N
CN CN CN
CN N
CN CNL
CNL CN CNL
non-NL
CN CN CN
non-NL TNL
non-NL
N*** NL
non-NL
K
