# Supplementary material for: The Eucalyptus grandis NBS-LRR Gene Family: Physical Clustering and Expression Hotspots
Source: Front Plant Sci. 2016 Jan 12;6:1238. doi: 10.3389/fpls.2015.01238 (PMC4709456; doi:10.3389/fpls.2015.01238)
Supplement: Figure S6 — NB-ARC-LRR fused domains (A) and TIR-NB-ARC-LRR fused domains (B). Conserved amino acid sequences are indicated with lines (top). The GKT (Kinase 1) conserved motif is recognized as a P-loop structure important in ATP hydrolysis while the hDD is also well conserved in NB-ARC domains (Kinase 2) as important in co-ordinating Mg2+ as a co-factor (Tameling et al., 2006). These two important sub-domains of NB-ARC are sometimes termed the Walker A and Walker B motifs (Walker et al., 1982) and are identified as A and B, respectively, within the I-Tasser protein structures (bottom) for a representative CNL (Eucgr.L01363) and TNL (Eucgr.C00020) sequence from the Eucalyptus grandis genome. [file Image6.PDF]

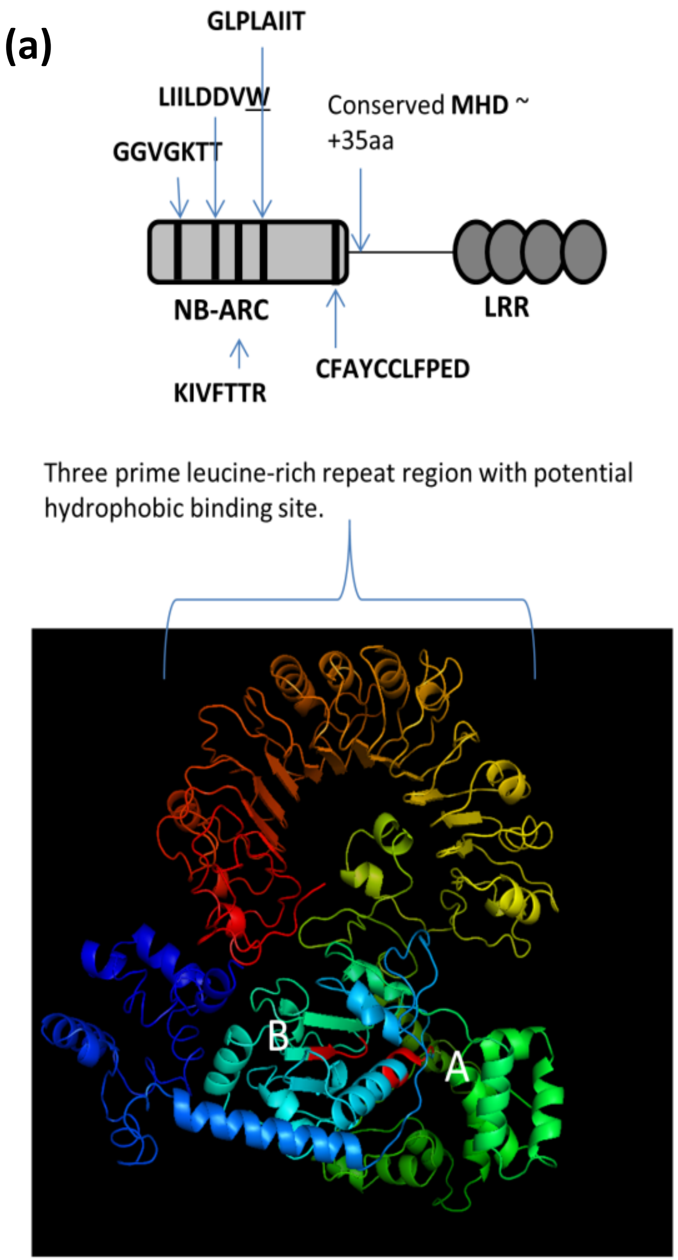

I-Tasser predicted structure for NBS-LRR (Eucgr.L01363). Kinase 1 (A) and Kinase 2 (B) motifs indicated. <u>W</u> –Tryptophan within Kinase 2.

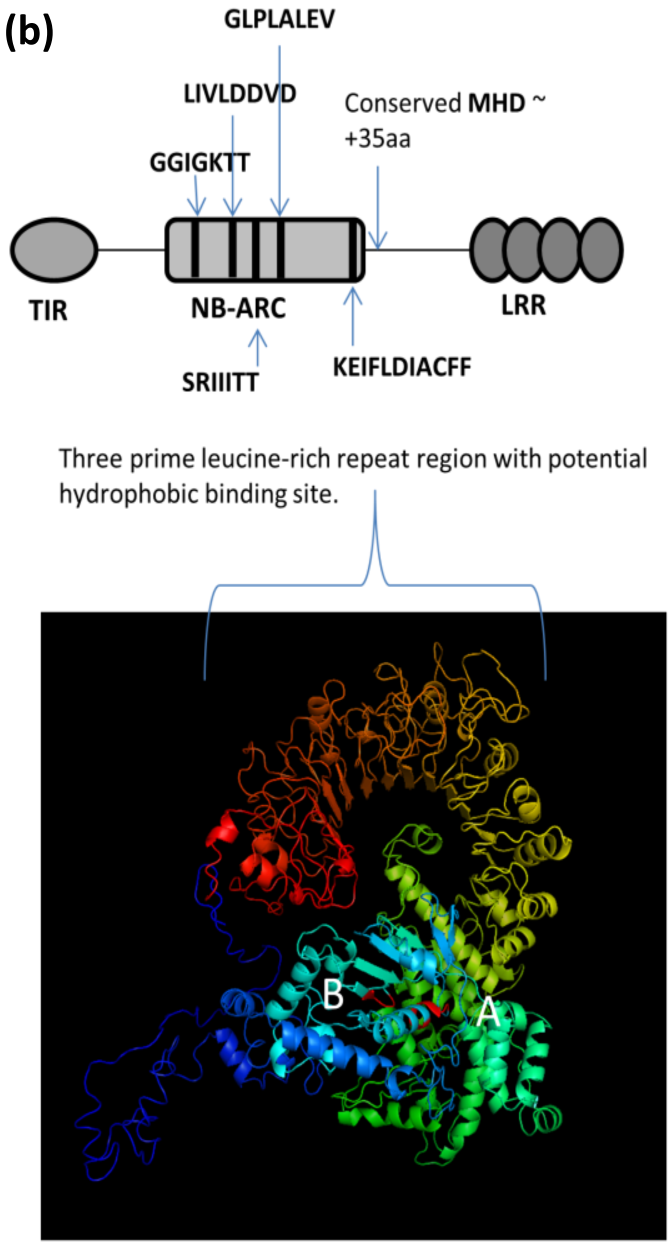

I-Tasser predicted structure for TIR-NBS-LRR (Eucgr.C00020). Kinase 1 (A) and Kinase 2 (B) motifs indicated.
